# The Interaction between Macrophages and Triple‐negative Breast Cancer Cells Induces ROS‐Mediated Interleukin 1α Expression to Enhance Tumorigenesis and Metastasis

**DOI:** 10.1002/advs.202302857

**Published:** 2023-08-08

**Authors:** Meng Hao, Bin Huang, Renfei Wu, Zheng Peng, Kathy Qian Luo

**Affiliations:** ^1^ Department of Biomedical Sciences Faculty of Health Sciences University of Macau Taipa Macao SAR 99078 China; ^2^ Ministry of Education Frontiers Science Center for Precision Oncology University of Macau Taipa Macao SAR 99078 China

**Keywords:** co‐culture, interleukin 1α (IL1α), macrophages, metastasis, reactive oxygen species (ROS), triple‐negative breast cancer (TNBC)

## Abstract

Triple‐negative breast cancer (TNBC) has higher mortality than non‐TNBC because of its stronger metastatic capacity. Increasing studies reported that TNBC tumors had more macrophage infiltration than non‐TNBC tumors, which promoted the metastasis of TNBC cells. However, how TNBC cells become more malignant after interacting with macrophages is less reported. In this study, it is observed that when TNBC cells are co‐cultured with macrophages, they display higher viability and stronger metastatic ability than non‐TNBC cells. Mechanistic studies reveal that TNBC cells acquired these abilities via interactions with macrophages in three phases. First, within 12 h of co‐culture with macrophages, some TNBC cells have significantly elevated levels of reactive oxygen species (ROS), which upregulate interleukin 1α (IL1α) expression in ERK1/2‐c‐Jun‐ and NF‐κB‐dependent manners at 24−48 h. Second, the secreted IL1α bound to IL1R1 activates the ERK1/2‐ZEB1‐VIM pathway which increases metastasis. Third, IL1α/IL1R1 facilitates its own synthesis and induces the expression of IL1β and IL8 at 72−96 h through the MKK4‐JNK‐c‐Jun and NF‐κB signaling pathways. Moreover, a higher level of IL1α is positively correlated with more macrophage infiltration and shorter overall survival in breast cancer patients. Thus, reducing ROS elevation or downregulating IL1α expression can serve as new strategies to decrease metastasis of TNBC.

## Graphical abstract

TNBC cells display higher viability than non‐TNBC cells and gain stronger metastatic abilities after being co‐cultured with macrophages. Macrophages induce ROS elevation and increase expression of IL1α, which is bound to IL1R1 to activate ERK1/2, MKK4, and NF‐κB‐mediated signaling pathways to facilitate tumor growth and metastasis. Reducing ROS elevation or neutralizing IL1α can serve as new strategies to decrease metastasis of TNBC.

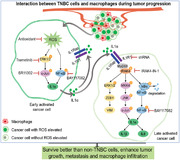

## 1 Introduction

The 5‐year mortality rate of patients diagnosed with triple‐negative breast cancer (TNBC) is 42%, which is much higher than the 28% average 5‐year mortality rate of patients with non‐TNBC.^[^ ^1^ ^]^ This high mortality rate is mainly caused by the stronger metastatic ability of TNBC cells.^[^ ^2^, ^3^ ^]^ For cancer cells to grow into a primary tumor and metastasize into secondary tumors, they must overcome the attack of immune cells, including macrophages, natural killer (NK) cells, and cytotoxic T lymphocytes. Among these three types of immune cells, macrophages are the only type that can change from type 1 cancer‐killing macrophages to type 2 cancer‐helping macrophages, which are often referred to as tumor‐associated macrophages (TAMs).^[^ ^4^, ^5^, ^6^ ^]^ Increasing evidence suggests that the crosstalk between cancer cells and macrophages has important implications for cancer metastasis.^[^ ^7^, ^8^ ^]^ Therefore, understanding how TNBC cells overcome macrophage attacks and utilize macrophages to acquire metastatic abilities is of great significance for the treatment of TNBC and for prolonging the lives of TNBC patients.

Previous studies have reported that cancer cells can use two mechanisms to avoid the recognition and killing of macrophages. The first mechanism is “do not eat me”, through which cancer cells express specialized cell surface proteins to avoid recognition and attack by macrophages. For example, some tumor cells can express high levels of immunoglobulin cluster of differentiation 47 (CD47),^[^ ^9^, ^10^, ^11^, ^12^ ^]^ which serve as a “do not eat me” signal by binding to the main ligand signal regulatory protein α (SIRPα) expressed on the surface of macrophages, thereby inhibiting macrophage‐mediated attacks.^[^ ^13^, ^14^ ^]^ Another “do not eat me” signal is a cluster of differentiation 24 (CD24), which is highly expressed in certain tumor cells and can interact with sialic acid‐binding immunoglobulin‐like lectin 10 (Siglec‐10) in macrophages thus avoiding macrophage engulfment.^[^ ^15^, ^16^ ^]^ Moreover, some tumor cells can express high levels of β2‐microglobulin (β2M), which can be attached to the surface of cancer cells by the major histocompatibility complex I (MHCI). The MHCI‐β2M complex can bind to leukocyte immunoglobulin‐like receptor B (LILRB) on the membrane of macrophages and inhibit the phagocytosis of the macrophages, thus escaping immune surveillance.^[^ ^17^, ^18^, ^19^ ^]^

The second mechanism is to polarize macrophages into the tumor‐helping M2 type with reduced killing abilities. For example, tumor cells can secrete cytokines, such as interleukin‐10 (IL‐10) and colony‐stimulating factor 1 (CSF‐1), to bind cell surface receptors on macrophages to polarize them toward the M2 phenotype thus reducing their phagocytotic effects.^[^ ^20^, ^21^ ^]^ Weng et al. found that the high expression of the oncogene multiple copies in T‐cell malignancy 1 (MCT‐1) in TNBC cells stimulated interleukin‐6 (IL‐6) secretion which transformed the human macrophages of the THP‐1 cells into the M2 type.^[^ ^22^ ^]^

Most previous studies regarding how cancer cells utilize macrophages to metastasize have focused on factors secreted from macrophages. For example, it has been reported that the high production of C‐C motif chemokine ligand 18 (CCL18) from TAMs promotes the invasion and metastasis of breast cancer xenografts via its functional receptor PITPNM family member 3 (PITPNM3).^[^ ^23^ ^]^ TAM‐secreted CCL18 can also form a positive feedback loop with CSF‐2 of cancer cells to induce epithelial‐to‐mesenchymal transition (EMT), cell migration, invasion, and metastasis of breast cancer cells.^[^ ^24^ ^]^ Moreover, Liu et al. reported that the high expression of LSECtin on TAMs interacts with the receptor butyrophilin subfamily 3 member A3 (BTN3A3) on cancer cells and enhances cancer stemness and growth of xenografted breast tumors.^[^ ^25^ ^]^ Animal experiments and clinical data also show that the high expression of various factors in TAMs, including lipocalin‐2 (LCN2), matrix metallopeptidase 9 (MMP9), macrophage inflammatory protein‐1β (MIP‐1β), chitinase‐3‐like protein 1 (CHI3L1), and cyclooxygenase‐2 (COX‐2), stimulate breast cancer cell migration and invasion.^[^ ^26^, ^27^, ^28^, ^29^, ^30^ ^]^

The aforementioned studies mainly focused on macrophage‐promoted metastatic effects. In this study, we investigated how cancer cells could drive the process of malignancy after interacting with macrophages. To achieve this goal, we co‐cultured various types of cancer cells with macrophages and found that the TNBC cells survived better than non‐TNBC cells when they encountered macrophages. More importantly, after being co‐cultured with macrophages, the TNBC cells increased their metastatic capacity. We then performed an RNA sequencing (RNA‐seq) analysis to identify the TNBC cell's driver genes that enabled the TNBC cells to survive when interacting with macrophages and gain stronger metastatic and tumor growth abilities. With these insights, we discovered a mechanism for the interaction between cancer cells and macrophages by which cancer cells actively acquire metastatic ability. This study may provide new therapeutic targets for preventing TNBC metastasis and treating malignant breast tumors.

## 2 Results

### 2.1 TNBC Cells Survived Better than non‐TNBC Cells when Co‐Cultured with Macrophages

To compare the viability between TNBC and non‐TNBC cells in the presence of macrophages, we co‐cultured three TNBC cell lines plus five non‐TNBC cells with Raw264.7‐tdT macrophages which express red fluorescent protein of tandem dimer tomato (tdT) in low‐adherence round‐bottom 96‐well plates for 7 days. The three TNBC cell lines included 231‐GFP and M1A‐C3, which were derived from MDA‐MB‐231 cells, and the 468‐clover cell line, which was derived from MDA‐MB‐468 cells. The five non‐TNBC cell lines included two estrogen receptor‐positive (ER+) breast cancer cell lines, MCF7‐C3 and T47D‐clover, one human epidermal growth factor receptor 2 positive (HER2+) breast cancer cell line, BT474‐clover, one lung cancer cell line, A549‐C3, and one cervical cancer cell line, HeLa‐C3.

The eight cancer cell lines express different fluorescent proteins including green fluorescent protein (GFP), clover which is a type of GFP, and sensor C3 which can detect caspase activation based on the principle of fluorescence resonance energy transfer (FRET). Sensor C3 was developed in our laboratory, and it constitutively expresses a fusion protein containing the cyan fluorescent protein (CFP), a linker containing the cleavage site of caspase 3/7, and the yellow fluorescent protein (YFP). Sensor C3 cells can emit green fluorescence when the cells are alive and emit blue fluorescence when caspase‐3 is activated during apoptosis.^[^ ^31^, ^32^, ^33^, ^34^ ^]^ In this study, the viability of the cancer cells were determined by measuring the intensities of green fluorescence from GFP, clover, or YFP.

**Figure 1:**
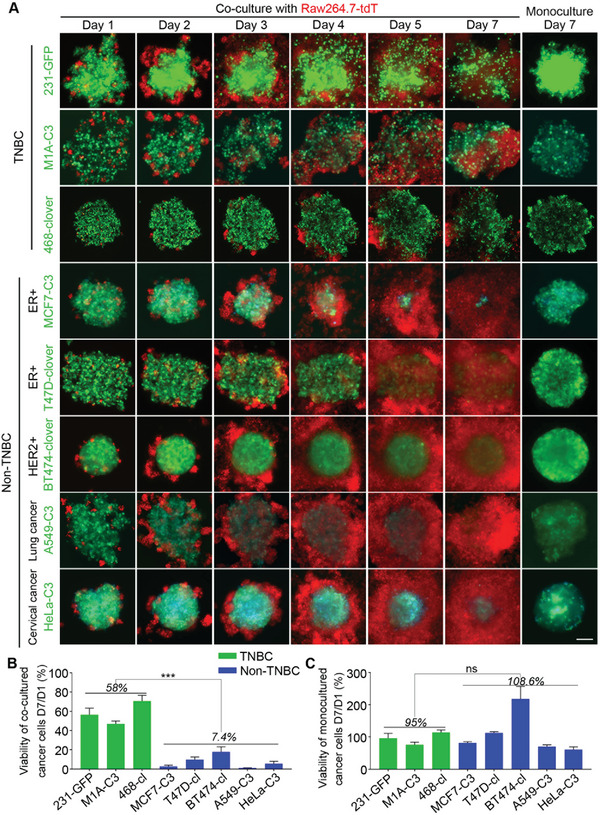
The TNBC cells survived better than the non‐TNBC cells when co‐cultured with macrophages. A) Representative images of the co‐cultured and monocultured cells. The three TNBC and five non‐TNBC cancer cell lines were co‐cultured with red‐fluorescent macrophages for 1−7 days. The initial co‐culture ratio of cancer cells and macrophages was 30:1, and the number of cancer cells was 2000. The sensor C3 cells expressed an apoptotic biosensor that emitted green fluorescence in live cells and blue fluorescence in apoptotic cells. B) The relative viabilities were calculated based on the fluorescence intensity of the cancer cells after being co‐cultured with macrophages on day 7 compared to their intensities on day 1. C) Quantified relative viabilities of monocultured cancer cells on day 7 compared to day 1 based on the green fluorescence intensity. The results represent the means ± SD from three independent experiments. Significant differences were determined by Student's t‐test. ***p < 0.001 and ns indicates no significance.

The fluorescence images revealed that many green‐colored TNBC cells remained after co‐cultured with macrophages, while very few non‐TNBC cells appeared green after 7 days of the co‐culture (**Figure** **1**A). The quantified viabilities of the three TNBC cell lines, M1A‐C3, 231‐GFP, and 468‐clover, were 47%, 56%, and 71%, respectively (Figure 1B). However, the viabilities of the five non‐TNBC cell lines, MCF7‐C3, T47D‐clover, BT474‐clover, A549‐C3, and HeLa‐C3 were much lower at 2.7%, 9.8%, 17.8%, 1.1%, and 5.5%, respectively (Figure 1B). In summary, the average viability of the three TNBC cell lines was 58%, while the average viability of five non‐TNBC cell lines was 7.4%, which was 7.8‐fold lower than that of the TNBC cells.

We also measured the ability of all eight cell lines to grow into three‐dimensional (3D) tumor spheres in non‐adhesive conditions. The micrographs showed that all the cell lines were able to grow into 3D tumor spheres within 7 days under monoculture conditions and the quantified results showed that there is no significant difference between the average viabilities of TNBC cells (95%) and non‐TNBC cells (108.6%) on day 7 compared to day 1 (Figure S1, Supporting Information; Figure 1A,C). These results indicate that TNBC cells have much higher viability than non‐TNBC cells when co‐cultured with macrophages.

In addition, we noticed that although the same number of macrophages was used at the beginning of co‐culture experiments, co‐culture with TNBC cells resulted in fewer macrophages than co‐culture with non‐TNBC cells on day 7 (Figure 1A and Figure S1, Supporting Information). These results suggest that TNBC cells may affect macrophage proliferation during the co‐culture.

### 2.2 The TNBC Cells Displayed Stronger Metastatic Phenotypes after Being Co‐cultured with Macrophages and had more Macrophages Infiltrated to the Tumor Sites

**Figure 2:**
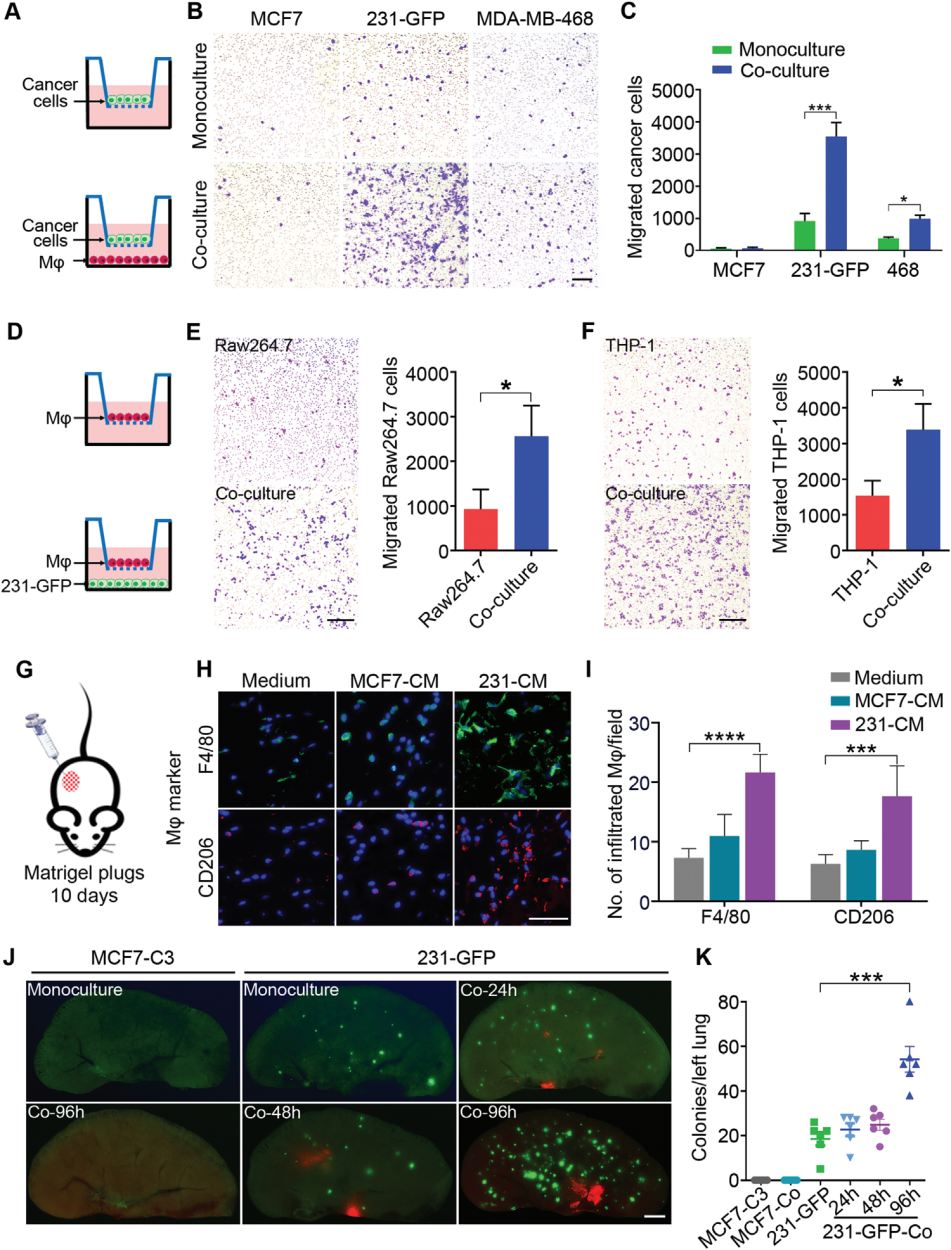
After being co‐cultured with the macrophages, TNBC cells displayed enhanced metastatic abilities and attracted more macrophages to the tumor sites. A) Schematic showing the design of a Transwell migration assay of MCF7, 231‐GFP, and MDA‐MB‐468 breast cancer cells. B,C) Representative images and the quantified number of migrated cancer cells under the mono‐ and co‐cultured conditions. D) Schematic diagram of the Transwell migration assay of the Raw264.7 and THP‐1 macrophages that can detect the tendency of macrophages to migrate toward cancer cells. The THP‐1 cells were previously stimulated with PMA (100 ng mL^−1^) for 24 h to differentiate into macrophages. E,F) Representative images and the quantified results of the Transwell migration assays for the Raw264.7 and THP‐1 macrophages. Scale bar, 200 µm. G) Schematic diagram of the Matrigel plug assay. H) Representative immunofluorescence images of F4/80 and CD206 in growth factor‐reduced Matrigel plugs supplemented with normal no‐serum DMEM or no‐serum conditioned medium of the MCF7 and 231‐GFP cells. Scale bar, 50 µm. I) Quantification of the F4/80^+^ or CD206^+^ macrophages in the Matrigel plugs. (*n* = 9 observation fields from 3 plugs). (J and K) Representative images and quantified numbers of green colonies per left lung of the mice injected with mono‐ or co‐cultured MCF7‐C3 and 231‐GFP cells through the tail vein for 28 days (*n* = 6 nude mice per group). Scale bar, 1 mm. The results represent the means ± SD from three independent experiments or from six mice. Significant differences were determined by Student's t‐test (C, E, F) or one‐way ANOVA (I, K). *p < 0.05, ***p < 0.001, and ****p < 0.0001.

With the results that the TNBC cells displayed higher viability than the non‐TNBC cells after being co‐cultured with macrophages, we further examined whether co‐culturing with macrophages could affect their metastatic properties. We designed a Transwell assay with cancer cells seeded into the upper chamber and macrophages seeded into the lower chamber after 96 h of co‐culture to detect the migration ability of the cancer cells (**Figure** **2**A). The results showed that the co‐culture with the macrophages significantly increased the migration ability of two TNBC cell lines, 231‐GFP and MDA‐MB‐468, by 2.9‐fold and 1.6‐fold, respectively, but no significant difference was observed between the monoculture and co‐cultured MCF7‐C3 cells (Figure 2B,C). These results showed that co‐culturing with macrophages for 96 h significantly elevated the metastatic abilities of the TNBC cells while co‐culturing with macrophages did not produce the same effects on the non‐TNBC cells.

Then, we used a Transwell migration assay to measure the migration ability of macrophages toward 231‐GFP cells (Figure 2D). The results showed that the presence of 231‐GFP cells in the lower chamber significantly enhanced the migration ability of both types of macrophages (Raw264.7 and THP‐1) by 2.7‐ and 2.2‐fold, respectively (Figure 2E,F).

We further used a Matrigel plug assay to determine whether the conditioned medium (CM) of TNBC and non‐TNBC cells could have different abilities to attract the host macrophages of mouse in vivo. We injected 200 µL of no‐serum CM from the MCF7 or MDA‐MB‐231 cells mixed with Matrigel at a 1:1 ratio into nude mice, no‐serum medium was used as a negative control and maintained the Matrigel plugs in mice for 10 days (Figure 2G). After that, the Matrigel plugs were fixed and stained with the macrophage marker F4/80 and M2 macrophage marker CD206 in immunofluorescence (IF) assay. The results showed that the no‐serum CM from the MDA‐MB‐231 cells significantly increased the number of F4/80‐ and CD206‐positive macrophages in the Matrigel plugs by 3.0‐ and 2.8‐fold, respectively, but there was no significant increase in Matrigel plugs containing the CM of the MCF7 cells (Figure 2H,I). These results suggest that TNBC cells might produce some factors to attract more macrophages and transform them into M2 macrophages during tumor progression.

We further compared the effects of TNBC and non‐TNBC cells on macrophage polarization. The qPCR results showed that co‐culture with TNBC (231‐GFP) cells increased the expression of M2 macrophage markers of CD163, CD204, and CD206 to 2.8‐ to 6.7‐fold compared to non‐TNBC (MCF‐7) cells. In contrast, co‐culture with non‐TNBC (MCF‐7) cells only enhanced the mRNA levels of M1 macrophage markers of TNFα, NOS2 and CD86 to 6.5‐ to 16.7‐fold compared to TNBC (231‐GFP) cells (Figure S2A, Supporting Information). These results indicate that TNBC cells can polarize macrophages to M2 type, while non‐TNBC cells can induce macrophages to differentiate into M1 type.

Next, we tested whether macrophages could also increase the metastatic ability of TNBC cells using a lung colony formation assay in mice. TNBC 231‐GFP and non‐TNBC MCF7‐C3 cells were separately co‐cultured with Raw264.7‐tdT macrophages for 24−96 h. Then, the mono‐ or co‐cultured cells were injected into the tail vein of nude mice, and the number of colonies in the left lung was determined by fluorescent microscopy after 28 days (Figure S2B, Supporting Information). For the 231‐GFP cells, compared with the monoculture treatment, the longer period of co‐culturing (96 h) significantly increased their lung colony‐forming ability by 2.9‐fold, while shorter periods of co‐culturing (24 and 48 h) did not have such effects. However, the MCF7‐C3 cells, even after a long period of co‐culturing (96 h), still could not form any colonies in the lungs of the mice (Figure 2J,K).

In addition, we observed that red fluorescent macrophages appeared in the lungs of the mice injected with the co‐cultured 231‐GFP cells and Raw264.7‐tdT macrophages, but no such phenomenon was observed in the mice injected with macrophages alone after they were monocultured or co‐cultured with 231‐GFP cells (Figure 2J, Figure S2C,D, Supporting Information). Additionally, no lung colonies were observed in the mice injected with co‐cultured MCF7‐C3 cells and macrophages (Figure 2J). These observations indicate that TNBC cells may have the ability to recruit macrophages and enhance their capacity to infiltrate into tumor sites in vivo.

Collectively, both the in vitro and in vivo results suggest that compared with non‐TNBC cells, TNBC cells survived better and acquired stronger metastatic abilities when co‐cultured with macrophages. TNBC cells could also attract more macrophages to the tumor sites, which might in turn further increase cancer metastasis.

### 2.3 Identification of the Key Genes that Were Differentially Expressed between the Monocultured and Co‐cultured TNBC Cells

**Figure 3:**
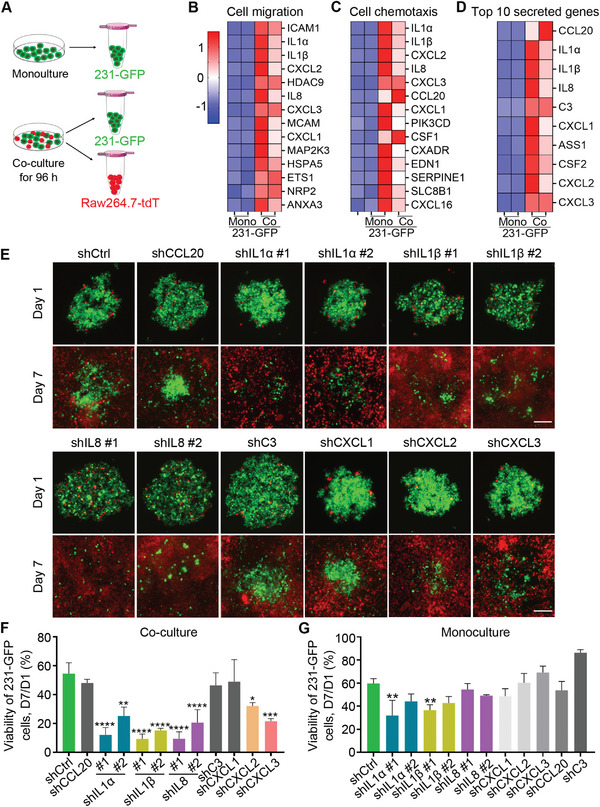
Identification of the driver genes that were up‐regulated in the co‐cultured TNBC cells. A) Schematic showing the RNA‐seq sample collection. B–D) Heatmap representation of the top 14 upregulated genes that are related to B) cell migration and C) cell chemotaxis, and D) the top ten upregulated secreted genes in the co‐cultured 231‐GFP cells. Red color indicates higher expression and blue color denotes lower expression. **E**) Representative images of tumor spheres formed by the 231‐GFP cells with or without the target gene knockdown when co‐cultured with Raw264.7‐tdT macrophages. Scale bar, 200 µm. F) Quantification of the total green fluorescence intensity indicating the viability of the co‐cultured and G) monocultured 231‐GFP cells on day 7 compared to day 1. The results represent the means ± SD from three independent experiments. Significant differences were determined by one‐way ANOVA. *p < 0.05, **p < 0.01, ***p < 0.001, and ****P < 0.0001.

To identify the driver genes of TNBC cells that enable them to survive, and acquire stronger metastatic ability after being co‐cultured with macrophages and recruit macrophages, RNA‐seq analysis was performed between the mono‐ and co‐cultured 231‐GFP cells (**Figure** **3**A). The results showed that 311 genes were significantly upregulated and 150 genes were significantly downregulated (*p* < 0.05) in the co‐cultured 231‐GFP cells (Figure S3A, Supporting Information). Gene Ontology (GO) enrichment analysis revealed that these genes are involved in multiple signaling pathways, including the inflammatory response, cell migration, and cell chemotaxis (Figure S3B, Supporting Information). Seven out of the top 22 genes involved in the pathways of cell migration and cell chemotaxis were among the top ten highly secreted genes. Three of the top five secreted genes were from the interleukin family (Figure 3B–D).

We designed human‐specific primers to amplify the top ten secreted genes by quantitative polymerase chain reaction (qPCR) in two TNBC cell lines, 231‐GFP and MDA‐MB‐468. The quantified qPCR results showed that eight of the top ten tested genes showed a 2−20‐fold elevation of mRNA levels after the co‐culture (Figure S3C,D, Supporting Information). Thus, we designed short hairpin‐mediated RNA (shRNA) to knockdown the expression of these eight genes (CCL20, IL1α, IL1β, IL8, C3, CXCL1, CXCL2, and CXCL3) and determined their importance in the observed phenotypes. The knockdown efficacies of these shRNAs were assessed in 231‐GFP cells by qPCR, and the results showed that at least one of the shRNAs significantly reduced the mRNA expression of target genes by >60% (Figure S3E, Supporting Information).

Next, we co‐cultured Raw264.7‐tdT macrophages and 231‐GFP cells transfected with gene‐specific shRNAs. The fluorescence images and the quantified results showed that reducing the expression of five out of the eight genes (IL1α, IL1β, IL8, CXCL2, and CXCL3) significantly reduced the viability of the 231‐GFP cells when they were co‐cultured with macrophages (Figure 3E,F). Among the five genes, only reducing the expression of IL1α and IL1β significantly reduced the viability of the 231‐GFP cells in the monoculture conditions, while knocking down the expression of the other three genes had no growth inhibitory effects (Figure 3G; Figure S4A, Supporting Information). These results indicate that these five candidate genes (IL1α, IL1β, IL8, CXCL2, and CXCL3) may help TNBC cells to survive when interacting with macrophages.

### 2.4 Determining the Driver Genes of TNBC Cells that Can Increase Cancer Metastasis and Macrophage Infiltration

**Figure 4:**
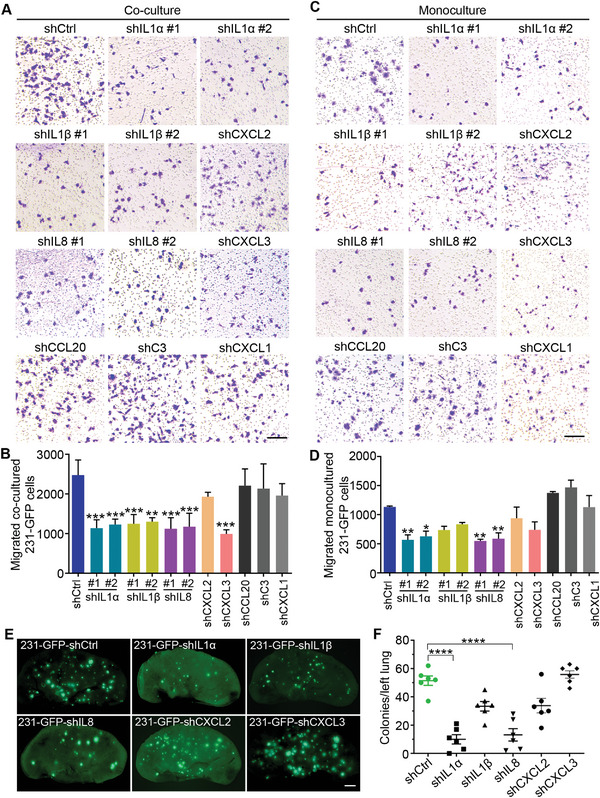
Determining the driver genes of TNBC cells that can increase cancer metastasis. A) Representative Transwell migration images of the 231‐GFP cells with or without the target gene knockdowns after being co‐cultured with Raw264.7 macrophages. Scale bar, 200 µm. B) The quantified number of the migrated 231‐GFP cells after the co‐culture. C) Representative images of the Transwell analysis of the monocultured 231‐GFP cells expressing gene‐specific shRNA. Scale bar, 200 µm. D) The quantified number of the migrated monocultured 231‐GFP cells. E,F) Representative fluorescence images of the lung and the quantified lung colony number of the 231‐GFP cells with or without the target gene knockdowns at 28 days after the tail vein injection in nude mice (*n* = 6 mice per group). Scale bar, 1 mm. The results represent the means ± SD from three independent experiments or from six mice. Significant differences were determined by one‐way ANOVA. *p < 0.05, **p < 0.01, ***p < 0.001, and ****p < 0.0001.

We subsequently investigated whether these candidate genes are important for TNBC cell migration, lung colony formation, and macrophage recruitment. The results of the Transwell migration assay showed that knockdown of IL1α, IL1β, IL8, and CXCL3 significantly reversed the enhanced migration ability of the 231‐GFP cells after they were co‐cultured with macrophages (**Figure** **4**A,B). However, knockdown of the expression of the other four genes, CXCL2, CCL20, C3, and CXCL1, did not achieve such effects (Figure 4A,B). We also measured the migration abilities of monocultured 231‐GFP cells modulated by respective shRNA, the results showed that only knockdown of IL1α and IL8 significantly reduced the number of migrated 231‐GFP cells under monoculture conditions (Figure 4C,D).

Then, we used a Transwell assay to assess the effects of knocking down the candidate genes on the recruitment ability of 231‐GFP cells on macrophages. The results showed that knockdown of IL1α, IL1β, IL8, and CXCL2 in the 231‐GFP cells significantly weakened their ability to attract Raw264.7‐tdT macrophages, but knockdown of CXCL3, CCL20, C3, and CXCL1 did not have such effects (Figure S4B–D, Supporting Information).

From the viability and migration results, we can conclude that five out of the eight candidate genes (IL1α, IL1β, IL8, CXCL2, and CXCL3) play important roles in increasing cancer cell survival and metastasis after being co‐cultured with macrophages, while the other three candidate genes (CCL20, C3, and CXCL1) did not show these effects. We then further tested the function of these five candidate genes in forming lung colonies in mice. TNBC 231‐GFP cells transfected with gene‐specific shRNAs were introduced into nude mice through tail vein injection. The fluorescence images and the quantified colony number showed that knockdown of IL1α and IL8 significantly reduced the lung colony formation of the 231‐GFP cells by 80.6% and 74.4%, respectively. Although knockdown of IL1β reduced the number of colonies in the lung by 35%, this reduction was statistically insignificant (Figure 4E,F). These results indicate that IL1α and IL8 may play more important roles than IL1β in promoting TNBC cell survival and the formation of lung metastatic colonies.

### 2.5 Knockdown of IL1α, IL1β and IL8 Reduces Tumor Growth, Orthotopically Derived Metastasis and Macrophage Infiltration to Tumor Sites

**Figure 5:**
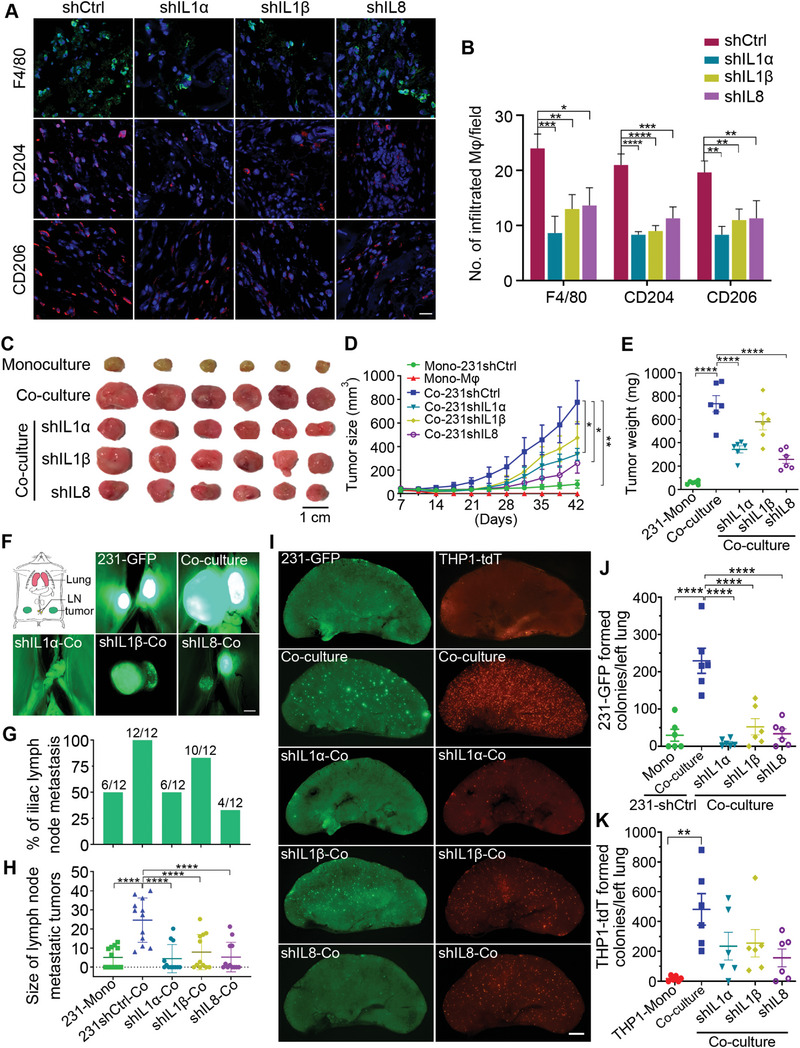
Knockdown of IL1α, IL1β and IL8 effectively reduced tumor growth and spontaneous metastasis in the co‐cultured 231‐GFP cells and decreased the infiltration of macrophages. A) Representative immunofluorescence images stained with macrophage marker F4/80, M2 macrophage markers of CD204 and CD206 in the Matrigel plugs supplemented with no‐serum conditioned medium of the 231‐GFP cells transfected with IL1α, IL1β or IL8 shRNAs. Scale bar, 20 µm. (*n* = 3 plugs). B) The quantified number of the F4/80+, CD204+, or CD206+ cells in each treatment group of the Matrigel plugs. (*n* = 9 observation fields from 3 plugs). C) Representative primary tumors 42 days after the implantation of the monocultured or co‐cultured 231‐GFP cells and THP1‐tdT macrophages into the mammary fat pad of NOD/SCID mice. (*n* = 6 mice per group). The 231‐GFP cells were transfected with shRNAs against IL1α, IL1β and IL8 or control shRNA. Scale bar, 1 cm. D) Primary tumor size was measured twice every seven days for 42 days. E) The weight of the primary tumors was determined on day 42. F) A schematic showing the anatomy of the primary tumors, nearby iliac lymph nodes (LN), and lung tissues of the mouse. Representative images of the iliac lymph node metastatic tumors of each treatment group were harvested on day 42. Scale bar, 1 mm. G) Percentage of lymphatic metastasis. H) Size of the lymph node metastatic tumors in each treatment group. I) Representative fluorescence images of the 231‐GFP cells and THP1‐tdT macrophages metastasized from the primary tumor to the lungs after they were injected into NOD/SCID mice 42 days prior. Scale bar, 1 mm. J) Quantification of the 231‐GFP cells‐derived lung colonies and K) THP1‐tdT macrophages‐derived lung colonies. The results represent the means ± SD from three independent experiments or from six mice. Significant differences were determined by two‐way ANOVA (D) and one‐way ANOVA (B,E,H,J,K). *p < 0.05, **p < 0.01, ***p < 0.001, and ****p < 0.0001.

To confirm the importance of IL1α, IL1β and IL8 in recruiting macrophages, we performed a Matrigel plug assay by injecting CM from the 231‐GFP cells transfected with gene‐specific or control shRNAs into nude mice and measured after 10 days. Fluorescence images and the quantified results showed that knockdown of the expression of IL1α, IL1β and IL8 significantly reduced the number of F4/80‐, CD204‐ and CD206‐positive macrophages that infiltrated into the Matrigel plugs by 42% to 64% (**Figure** **5**A,B).

To compare the primary tumorigenic and spontaneous metastatic ability between the monoculture and co‐cultured TNBC cells. We co‐cultured 231‐GFP cells and human‐derived THP1‐tdT macrophages for 96 h. And then inoculated the monocultured and co‐cultured cells into the mammary fat pad in 6‐week‐old NOD/SCID mice, which is an orthotopic tumor model for breast cancer. The results showed that the monocultured 231‐GFP cells formed much smaller tumors, while the co‐cultured 231‐GFP cells formed much larger tumors with the tumor size and tumor weight increasing by 9.6‐ and 12.5‐fold, respectively (Figure 5C–E).

To determine the roles of IL1α, IL1β and IL8 in promoting tumor growth, we knocked down the expression of these three genes using shRNAs and measured the tumor size after 42 days. The results showed that reducing the expression of IL1α or IL8 significantly reduced the tumor size of the 231‐GFP cells co‐cultured with THP1‐tdT macrophages by 57% and 67%, respectively, and decreased the tumor weight by 53% and 65%, respectively (Figure 5C–E). Similar to the results obtained from the lung colony formation experiment, knockdown of IL1β in the 231‐GFP cells also had less effects than knockdowns of IL1α and IL8, resulting in the reduction of the tumor size by 39% and tumor weight by 21% (Figure 5C–E). The body weights of the animals in the different treatment groups did not show significant changes at six weeks (Figure S5, Supporting Information).

Using a spontaneous metastatic tumor model, we found that 231‐GFP cells co‐cultured with THP1‐tdT macrophages significantly increased the number of mice bearing metastatic tumors in iliac lymph nodes by 2‐fold (from 50% to 100%) and increased the size of these lymphatic tumors by 4.8‐fold (Figure 5F–H). Furthermore, after the co‐culture, the average number of 231‐GFP‐derived lung metastatic colonies significantly increased from 29.8 in the monoculture to 229.3 in the co‐culture which was a 7.7‐fold increase (Figure 5I,J).

Knockdown of IL1α and IL8 decreased the percentage of lymphatic metastasis from 100% to 50% and 33%, respectively, which was more effective than the reduction rate of shIL1β (83%) (Figure 5F,G). More importantly, knockdown of the expression of IL1α, IL1β and IL8 significantly reduced the size of metastatic tumors in the iliac lymph nodes by 81.9%, 68.0%, and 78.5%, respectively (Figure 5F,H). The number of metastatic colonies in the lungs was also significantly reduced by 96.1%, 77.1%, and 85.3%, respectively (Figure 5I,J). Among the three cytokines, decreasing the expression of IL1α seems to produce the highest reductive effects on cancer metastasis.

In addition to increasing the metastatic ability of the cancer cells, the co‐culture treatment also increased the number of macrophages infiltrating the lung from 17.5 to 481.8, which was a 27.5‐fold elevation (Figure 5I,K). Knockdown of IL1α, IL1β and IL8 reduced the number of infiltrated macrophage colonies from 481.8 to 234.8, 254.8, and 156.7, respectively, which is an ≈47.1% to 67.5% reduction (Figure 5I,K). Collectively, these results indicate that IL1α, IL1β and IL8 are necessary for 231‐GFP cells to interact with macrophages and subsequently gain the abilities of tumor growth, cancer metastasis, and macrophage infiltration of the tumor sites.

### 2.6 Co‐Culturing Induced ROS Elevation and Activated the ERK1/2‐c‐Jun and NF‐κB Signaling Pathways to Upregulate the Expression of IL1α, IL1β and IL8

**Figure 6:**
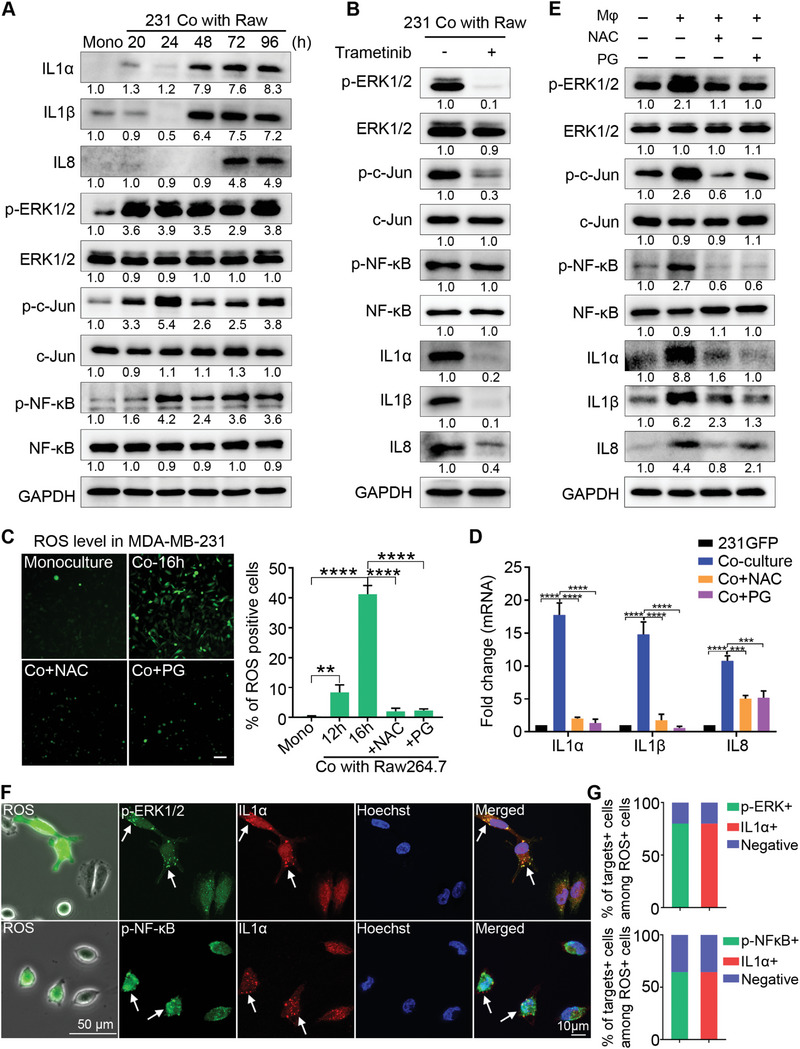
Co‐culturing induced ROS elevation and activated the ERK1/2‐c‐Jun and NF‐κB signaling pathways to upregulate the expression of IL1α, IL1β and IL8. A) Western blotting of the total or phosphorylated protein levels of IL1α, IL1β, IL8, ERK1/2, c‐Jun, and NF‐κB in the mono‐ or co‐cultured 231‐GFP cells with Raw264.7‐tdT. B) Western blotting of ERK1/2, p‐ERK1/2, c‐Jun, p‐c‐Jun, NF‐κB, p‐NF‐κB, IL1α, IL1β and IL8 in the 231‐GFP cells treated with or without the MEK inhibitor trametinib (10 nM) during 96 h of the co‐culture treatment with Raw264.7‐tdT. The medium containing the inhibitor was replaced every two days. C) (left) DCFDA‐stained fluorescence images of the mono‐ or 16 h co‐cultured MDA‐MB‐231 cells with Raw264.7, with or without the NAC (5 mM) and PG (20 µM) treatment. Scale bar, 100 µm. (right) Quantitative results of the percentage of ROS‐positive MDA‐MB‐231 cells. D) qPCR showing the mRNA levels of IL1α, IL1β and IL8 in the mono‐ or co‐cultured 231‐GFP cells with Raw264.7‐tdT, with or without the NAC (5 mM) and PG (20 µM) treatment during co‐culturing. E) Western blotting results showing the total or phosphorylated protein levels of ERK1/2, c‐Jun, NF‐κB, IL1α, IL1β and IL8 in the mono or co‐cultured 231‐GFP cells with or without NAC (5 mM) and PG (20 µM) during 96 h of the co‐culture treatment with Raw264.7‐tdT. F) Fluorescence and phase images of the MDA‐MB‐231 cells after 48 h of co‐culturing with Raw264.7 followed by DCFDA staining for ROS detection (left column). Scale bar, 50 µm. Representative co‐IF staining images of the distribution of p‐ERK1/2 and IL1α or p‐NF‐κB and IL1α in the same cells that were stained with DCFDA. Scale bar, 10 µm. G) Percentages of the cells stained with higher levels of both p‐ERK1/2 and IL1α or p‐NF‐κB and IL1α among the ROS‐positive MDA‐MB‐231 cells. (*n* = 50 cells). The results represent the means ± SD from three independent experiments. Significant differences were determined by one‐way ANOVA. **p < 0.01, ***p < 0.001 and ****p < 0.0001.

To elucidate the possible mechanisms that resulted in the upregulation of IL1α, IL1β and IL8 after the co‐culture, we used Western blot analysis to determine the changes in the protein levels at different times during the co‐culture treatment. For IL1α and IL1β, their protein levels were greatly increased at 48 h, while the level of IL8 was increased later at 72 h of the co‐culture treatment (**Figure** **6**A). We observed that the levels of phosphorylated extracelluar signal‐regulated protein kinases 1 and 2 (p‐ERK1/2), phosphorylated c‐Jun (p‐c‐Jun), and phosphorylated nuclear factor kappa‐light‐chain‐enhancer of activated B cells (p‐NF‐κB) increased 3.3‐ to 4.2‐fold between 20 and 24 h of the co‐culture treatment (Figure 6A), which is much earlier than the increased expression of IL1α, IL1β and IL8 at 48 to 72 h. The protein levels of phosphorylated dual‐specificity mitogen‐activated protein kinase kinase 4 (p‐MKK4) and phosphorylated c‐Jun N‐terminal kinase (p‐JNK) increased at 48 and 72 h of the co‐culture treatment, which were at the same time points as the upregulation of IL1α, IL1β and IL8 (Figure S6A, Supporting Information).

Subsequently, we inhibited the activation of p‐ERK1/2 using a mitogen‐activated protein kinase kinase (MEK) inhibitor (trametinib) during the co‐culture and found that the MEK inhibitor greatly reduced the protein levels of p‐ERK1/2, p‐c‐Jun, IL1α, and IL1β and partially reduced the level of IL8 but had no effect on the level of p‐NF‐κB (Figure 6B). Moreover, the application of inhibitors of c‐Jun (with SR11302) and NF‐κB (with BAY117082) only partially reduced the levels of IL1α, IL1β and IL8 after the co‐culture treatment (Figure S6B,C, Supporting Information). These results suggest that ERK1/2 activation is an early event during co‐culturing and plays an important role in upregulating the expression of IL1α, IL1β and IL8.

A previous study showed that ERK1/2 could be activated when the intracellular levels of reactive oxygen species (ROS) were elevated by circulatory treatment in TNBC MDA‐MB‐231 cells.^[^ ^35^ ^]^ Therefore, we investigated whether ROS could be generated during a separate co‐culture of MDA‐MB‐231 with Raw264.7 by using a fluorescent dye of CM‐H2DCFDA (DCFDA) to stain the cancer cells. The fluorescence images and the quantified results showed that in the monoculture, very few cells had ROS; while in the co‐culture, the percentage of ROS‐positive cells increased from 8.7% at 12 h to 41.2% at 16 h (Figure 6C, Figure S6D,E, Supporting Information). The significant increases in the ROS levels during the co‐culture were completely prevented by the addition of two antioxidants, *N*‐acetyl‐cysteine (NAC) and propyl gallate (PG) (Figure 6C). Importantly, treatment with the antioxidants NAC and PG during the co‐culture dramatically reduced the mRNA levels of IL1α and IL1β, but partially decreased the mRNA level of IL8 in the 231‐GFP cells (Figure 6D). These two antioxidants produced similar reductive effects on the protein expression of the three cytokines and inhibited the phosphorylation of ERK1/2, c‐Jun, and NF‐κB (Figure 6E).

We then investigated whether the elevation of ROS was correlated with the upregulation of p‐ERK1/2, p‐NF‐κB and IL1α at the single‐cell level. MDA‐MB‐231 cells were seeded in the lower chamber of the Transwell insert with a gridded glass coverslip for easy tracking of their localization, and macrophages were added to the upper chamber of the Transwell insert. After 48 h of the co‐culture treatment, the MDA‐MB‐231 cells were stained with ROS dye (DCFDA). The location and intensity of the ROS‐positive cells were obtained by phase and fluorescence microscopy. Afterward, the cells were fixed and co‐immunostained with ERK1/2 plus IL1α antibodies or NF‐κB plus IL1α antibodies. The fluorescence images and the quantified results showed that 80% of the ROS‐positive cells displayed stronger fluorescence signals for both p‐ERK1/2 and IL1α. A slightly lower percentage of ROS‐positive cells (64.5%) exhibited higher fluorescence signals for both IL1α and p‐NF‐κB. We noticed that the majority of the ROS‐positive cells had high levels of p‐NF‐κB localized in the nucleus (Figure 6F,G).

Furthermore, treating the MDA‐MB‐231 cells with 100 µM hydrogen peroxide (H_2_O_2_) for 6 h not only greatly elevated the intracellular levels of ROS but also significantly increased the mRNA and protein levels of IL1α, IL1β and IL8 (Figure S6F–H, Supporting Information). Pre‐treating MDA‐MB‐231 cells with antioxidants NAC or PG prevented the elevation of H_2_O_2_‐induced ROS and the upregulation of IL1α, IL1β and IL8 (Figure S6F–H, Supporting Information).

To test whether macrophages could also induce the elevation of ROS in non‐TNBC cells, we measured ROS levels after non‐TNBC MCF‐7 cells were cultured with or without macrophages. The results showed that the ROS level was increased 7.1‐fold in MCF‐7 cells after co‐cultured with Raw264.7 macrophages (Figure S6I, Supporting Information). However, co‐culturing with macrophages did not significantly elevate the mRNA and protein levels of IL1α, IL1β and IL8 in MCF‐7 cells compared with monocultured MCF‐7 cells (Figure S6J,K, Supporting Information). These results suggest that although macrophages could induce ROS elevation in both TNBC and non‐TNBC cells, the elevated ROS could only trigger the production of IL1α, IL1β and IL8 in TNBC cells but not in non‐TNBC cells.

Taken together, we propose that the co‐culture treatment elevates the levels of ROS in the TNBC cells, which then activates the ERK1/2‐c‐Jun and NF‐κB signaling pathways to increase the expression of IL1α, IL1β and IL8.

### 2.7 IL1α Activated Three Signaling Pathways Mediated by ERK1/2, MKK4 and NF‐κB in Co‐Cultured TNBC Cells Via its Receptor IL1R1

IL1α transduces its intracellular signaling cascades by binding to its receptor IL1R1. To determine the importance of IL1R1 in promoting co‐culture‐induced tumor growth and cancer metastasis, we used shRNA to knockdown the expression of IL1R1 in 231‐GFP cells. Both qPCR and Western blot results showed that shIL1R1 reduced the expression of IL1R1 by over 80% (Figure S7A,B, Supporting Information). The knockdown of IL1R1 also reduced the viability of the 231‐GFP cells by 60% (Figure S7C, Supporting Information) and significantly decreased the migration of the 231‐GFP cells by 61.9% during the co‐culture (Figure S7D, Supporting Information).

**Figure 7:**
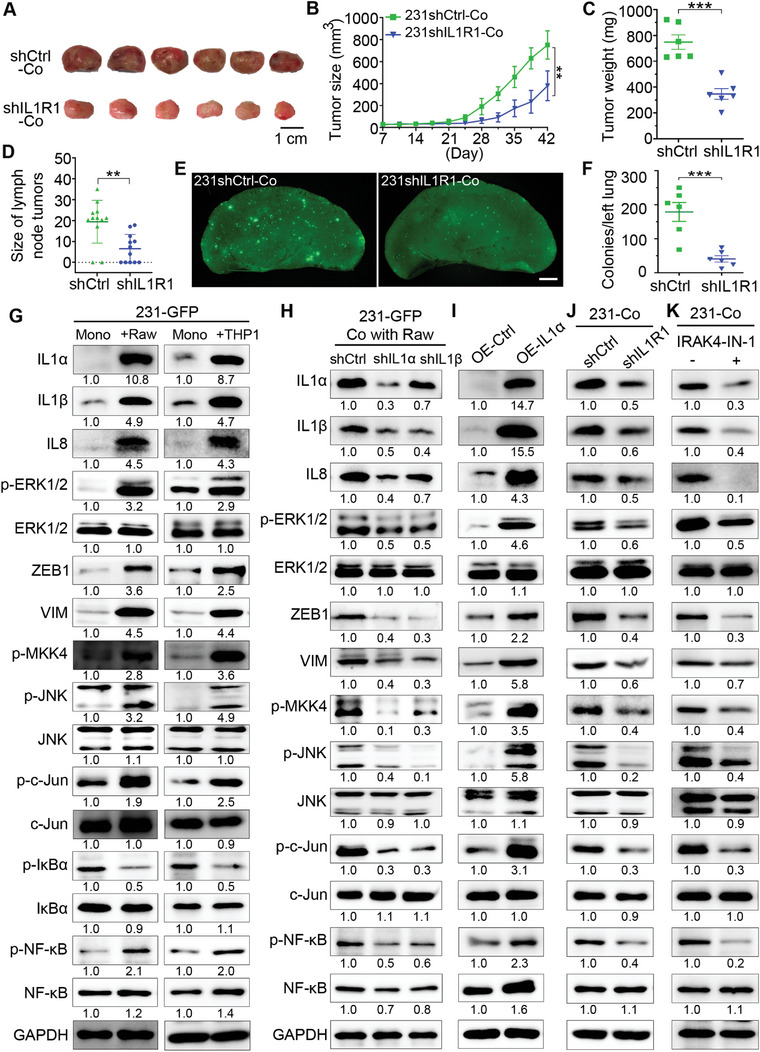
IL1α activated three signaling pathways mediated by ERK1/2, MKK4, and NF‐κB in co‐cultured TNBC cells via its receptor IL1R1. A) Representative primary tumors 42 days after implantation of the co‐cultured 231‐GFP cells and THP1‐tdT macrophages into the mammary fat pad of NOD/SCID mice. (*n* = 6 mice per group). The 231‐GFP cells were transfected with IL1R1 shRNA or control shRNA. Scale bar, 1 cm. B) Primary tumor growth of each treatment group, which was monitored twice every 7 days for 42 days. C) Primary tumor weight of each treatment group on day 42. D) The size of the lymph node metastatic tumors of each treatment group. E) Representative lung images of the NOD/SCID mice harvested on day 42. Scale bar, 1 mm. F) Quantification of the lung colonies metastasized from primary tumors of each treatment group. G) Western blots showing the total or phosphorylated protein levels of IL1α, IL1β, IL8, ERK1/2, ZEB1, VIM, MKK4, JNK, c‐Jun, IκBα and NF‐κB in 231‐GFP cells before and after 96 h of the co‐culture treatment with Raw264.7‐tdT or THP1‐tdT macrophages. H) Western blots showing the knockdown effects of IL1α and IL1β on themselves and other indicated proteins in 231‐GFP cells co‐cultured with Raw264.7‐tdT cells for 96 h. I) Western blots showing the effects of overexpressing IL1α on itself and other indicated proteins in 231‐GFP cells. J) Western blots showing the expression levels of the indicated proteins in the co‐cultured 231‐GFP cells with the knockdown of IL1R1. K) Western blotting of the indicated proteins in co‐cultured 231‐GFP cells with or without the treatment with the IRAK4 inhibitor (IRAK4‐IN‐1, 5 µM) during co‐culturing. The results represent the means ± SD from three independent experiments or from six mice. Significant differences were determined by two‐way ANOVA (B) or Student's t‐test (C,E). **p < 0.01, ***p < 0.001.

Animal results showed that knockdown of IL1R1 significantly reduced the tumor size by 49% and the tumor weight by 53.6% of co‐cultured 231‐GFP cell‐derived xenograft tumors in NOD/SCID mice (**Figure** **7**A–C). There was no difference between the average body weights of the mice injected with 231GFP‐shIL1R1 and 231GFP‐shCtrl cells (Figure S7E, Supporting Information). The knockdown of IL1R1 also reduced the occurrence of lymphatic metastasis by 25%, significantly decreased the size of lymphatic metastatic tumors by 66.4%, and reduced the number of lung metastatic colonies by 77.2% after 42 days of animal experiments (Figure 7D–F; Figure S7F,G, Supporting Information).

To determine the downstream signaling pathways activated by IL1α/IL1R1 in TNBC cells during the co‐culture, we first validated that co‐culturing TNBC 231‐GFP cells with either mouse or human macrophages or co‐culturing another TNBC MDA‐MB‐468 cells with these two types of macrophages, greatly increased the expression of IL1α, IL1β and IL8 (Figure 7G; Figure S8A, Supporting Information). The Western blot results showed that the co‐culture treatment also increased the total or phosphorylated levels of multiple proteins involved in three signaling pathways in both TNBC cell lines. 1) ERK1/2‐zinc finger E‐box binding homeobox 1 (ZEB1)‐vimentin (VIM); 2) MKK4‐JNK‐c‐Jun; 3) IκBα‐NF‐κB (Figure 7G; Figure S8A, Supporting Information). The protein levels of total and phosphorylated Akt and p38 did not increase in the co‐cultured 231‐GFP cells, indicating that Akt‐ and p38‐mediated signaling pathways were not activated by IL1α during the co‐culturing (Figure S8B, Supporting Information).

To determine the relationships between IL1α, IL1β and IL8, we knocked down each of the three cytokines and measured their mRNA levels in monocultured 231‐GFP cells. The qPCR results showed that knockdown of IL1α significantly reduced the mRNA levels of both IL1β and IL8, while knockdown of IL1β or IL8 did not significantly affect the mRNA level of IL1α (Figure S7H–J, Supporting Information). Western blot results also confirmed that knockdown of the expression of IL1α reduced the protein levels of IL1β and IL8 by 50–60%, while shIL1β only reduced the protein levels of IL1α and IL8 by 30% (Figure 7H), which further validates that the function of IL1α is upstream of IL1β and IL8 during the co‐culturing. The knockdown of either IL1α or IL1β reduced the levels of p‐ERK1/2, ZEB1, VIM, p‐MKK4, p‐JNK, p‐c‐Jun and p‐NF‐κB (Figure 7H and Figure S8C, Supporting Information). In addition, the overexpression of IL1α in the 231‐GFP cells increased the total protein levels of IL1β, IL8, ZEB1, VIM, and phosphorylated protein levels of ERK1/2, MKK4, JNK, c‐Jun, and NF‐κB (Figure 7I).

Next, we also checked the effects of knocking down the receptor of IL1α on its downstream targets and found that shIL1R1 reduced the protein levels of IL1α, IL1β, IL8, p‐ERK1/2, ZEB1, VIM, p‐MKK4, p‐JNK, p‐c‐Jun and p‐NF‐κB (Figure 7J; Figure S8D, Supporting Information). We also inhibited interleukin‐1 receptor‐associated kinase 4 (IRAK4) activation with its inhibitor (IRAK4‐IN‐1) in co‐cultured 231‐GFP cells to verify whether IL1α can activate its downstream signaling pathways through IL1R1/IRAK4. The results showed that IRAK4‐IN‐1 reduced the protein levels of IL1α, IL1β, IL8, p‐ERK1/2, ZEB1, VIM, p‐MKK4, p‐JNK, p‐c‐Jun and p‐NF‐κB (Figure 7K; Figure S8E, Supporting Information).

Our previous study showed that ERK1/2 can activate ZEB1 and VIM to increase metastasis in lung cancer cells.^[^ ^36^ ^]^ In this study, we added a MEK inhibitor to co‐cultured 231‐GFP cells and found that the levels of ZEB1 and VIM were greatly reduced (Figure S8F, Supporting Information), which suggested that ERK1/2 acts upstream of ZEB1 and VIM. Time course analysis also showed that the protein level of ZEB1 was elevated at 48 h and that of VIM was increased at 96 h of co‐culture (Figure S8G, Supporting Information), which is much later than the time of ERK1/2 activation at 20 h (Figure 6A).

### 2.8 Overexpression of IL1α Enhanced Tumor Growth, Cancer Metastasis and Macrophage Infiltration

To validate the function of IL1α in promoting tumor growth, cancer metastasis, and macrophage recruitment, we overexpressed IL1α in the 231‐GFP cells and assessed its effects in vitro and in vivo. The qPCR results showed that overexpressing IL1α not only significantly increased its own mRNA levels, but also greatly enhanced the mRNA levels of IL1β and IL8 (Figure S9A, Supporting Information). The overexpression of IL1α significantly increased the viability of the 231‐GFP cells by 70% under 3D co‐cultured conditions (Figure S9B,C, Supporting Information). The overexpression of IL1α also significantly increased the number of migrated and invaded 231‐GFP cells by 2.1‐ and 2.3‐fold, respectively (Figure S9D,E, Supporting Information). Moreover, high expression of IL1α significantly increased the lung colony numbers of the 231‐GFP cells (2.0‐fold) in the nude mice injected with cancer cells through tail vein injection (Figure S9F,G, Supporting Information).

**Figure 8:**
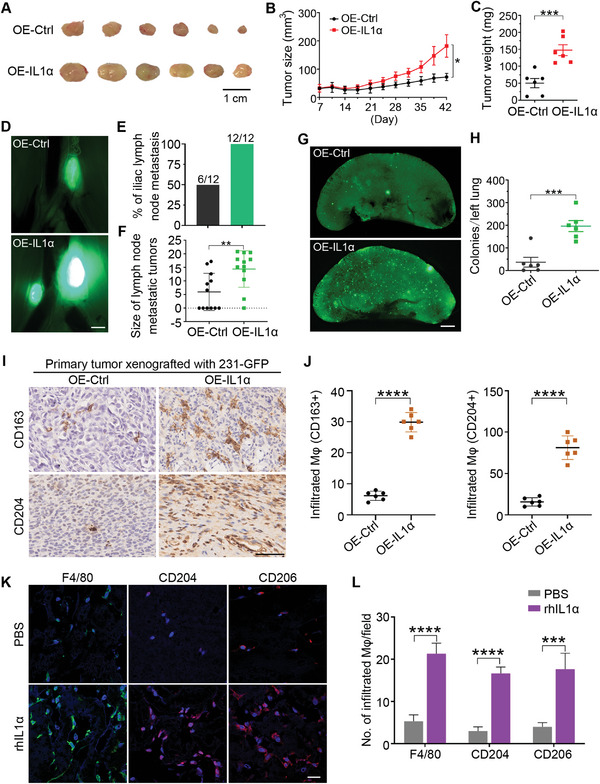
Overexpression of IL1α enhanced tumor growth, cancer metastasis, and macrophage infiltration into xenografted tumors. A) Representative primary tumors 42 days after implanting the 231‐GFP cells expressing the empty vector (OE‐Ctrl) or IL1α (OE‐IL1α) into the mammary fat pad of NOD/SCID mice (*n* = 6 mice per group). Scale bar, 1 cm. B) Primary tumor size was measured twice every seven days for 42 days (*n* = 6 mice per group). C) The weight of the primary tumors from six mice per group was determined on day 42. D) Representative images of the iliac lymph node metastatic tumors of each treatment group were harvested on day 42 (*n* = 6 mice per group). Scale bar, 1 mm. E) Percentage of lymphatic metastasis in each treatment group. F) Size of the lymph node metastatic tumors in each treatment group. G) Representative fluorescence images of the 231‐GFP cell‐derived colonies metastasized from the primary tumor to the lung after they were injected into NOD/SCID mice 42 days prior. Scale bar, 1 mm. H) Quantification of the lung colonies metastasized from the primary tumors of each treatment group. I) Representative IHC staining of the M2 macrophage markers CD163 and CD204 from the 231‐GFP cell‐derived xenograft tumors. Scale bar, 50 µm. J) Quantification of the number of infiltrated macrophages in the tumor section. K) Representative immunofluorescence images stained with F4/80, CD204, and CD206 in the Matrigel plugs supplemented with PBS or human recombinant IL1α protein. Scale bar, 20 µm. (*n* = 3 plugs per group). L) The quantified number of F4/80+, CD204+, and CD206+ cells in each treatment group of the Matrigel plugs. (*n* = 9 observation fields from 3 plugs). The results represent the means ± SD from three independent experiments or from six mice. Significant differences were determined by two‐way ANOVA (B) or Student's t‐test (C,F,H,J,L). *p < 0.05, **p < 0.01, ***p < 0.001, and ****p < 0.0001.

In vivo animal results showed that overexpressing IL1α increased the size and weight of the 231‐GFP‐derived xenograft tumors by 1.5‐ and 1.9‐fold, respectively (**Figure** **8**A–C). And overexpressing IL1α increased the percentage of iliac lymph node metastasis of the xenografted tumors from 50% to 100% and significantly increased the size of the iliac lymph node metastatic tumors by 1.4‐fold (Figure 8D–F). Overexpressing IL1α also increased the spontaneous lung metastasis by 4.3‐fold from 36.8 to 196.3 colonies (Figure 8G,H).

More importantly, immunohistochemical (IHC) staining showed that the overexpression of IL1α significantly increased the infiltration of CD163+ or CD204+ macrophages into the xenografted tumor tissues by 4.8‐ and 5.1‐fold, respectively (Figure 8I,J). Similarly, subcutaneously implanting Matrigel plugs containing recombinant human IL1α protein attracted 4.0‐ to 5.6‐fold more F4/80+, CD204+, and CD206+ macrophages into the Matrigel plugs (Figure 8K,L). Taken together, these results revealed that overexpression of IL1α enhanced tumor growth, cancer metastasis, and macrophage infiltration into xenografted tumor sites.

### 2.9 High Expression of IL1α Correlated with Shorter Survival Time and more M2 Macrophage Infiltration in Breast Tumors of TNBC Patients

We then used the TCGA data source in the TIMER 2.0 platform to conduct a metadata analysis to determine the association between the expression of these three cytokines and the overall survival of breast cancer patients. The results showed that higher expressions of IL1α and IL1β, but not IL8, are significantly correlated with shorter overall survival times for breast cancer patients (Figure S9H, Supporting Information).

Moreover, high levels of the M2 macrophage marker CD163, but not CD204 significantly correlated with a shorter survival time in patients with breast cancer (Figure S9I, Supporting Information, left and middle panels). In addition, analysis of the association between immune infiltration and the clinical outcome showed that high M2 macrophage infiltration negatively correlated with the overall survival of breast cancer patients (Figure S9I, Supporting Information, right panel).

**Figure 9:**
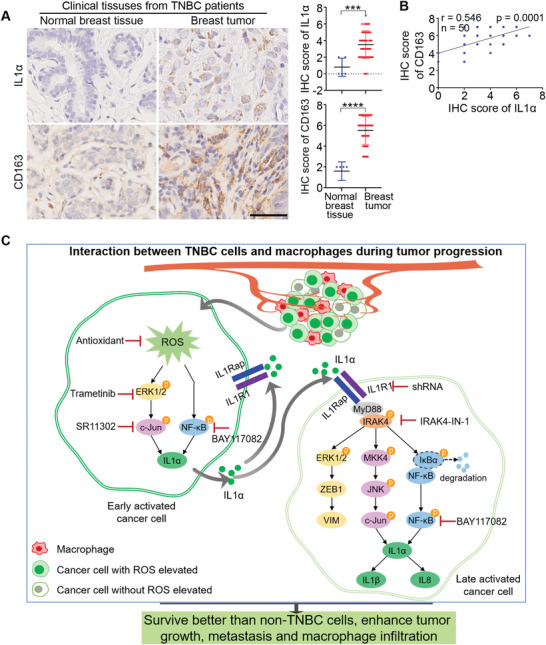
High expression of IL1α correlated with shorter survival time and more M2 macrophage infiltration in breast tumors of TNBC patients. A) Representative IHC images of IL1α and the M2 macrophage marker CD163 in paraffin sections of normal breast tissues (*n* = 5) and breast tumors from TNBC patients (*n* = 50). Scale bar, 50 µm. Graphs showing the IHC scores of IL1α and CD163 between normal breast tissues and TNBC breast tumors. Each dot represents one sample. IHC staining intensities were assessed by a semiquantitative system according to the immunoreactive score. B) The positive correlation between the IHC score of IL1α and that of CD163 in the patients with TNBC (*n* = 50). C) Proposed signaling pathways depicting the mechanisms by which TNBC cells survive better than non‐TNBC and acquire stronger tumor growth and cancer metastasis abilities when interacting with macrophages. Significant differences were determined by Student's t‐test (A). *** *p* < 0.001, ****p < 0.0001.

To further evaluate the clinical relevance of IL1α expression and macrophage infiltration in patients with TNBC, we used IHC staining to compare the levels of IL1α and CD163 between normal breast tissues and breast tumors. The images and the quantified results showed that the levels of IL1α and CD163 were significantly higher in the TNBC tumors than in the normal breast tissues (**Figure** **9**A). Moreover, there was a positive correlation between the IHC score of IL1α and that of CD163 (Figure 9B). This positive correlation suggest that a high expression of IL1α increased the infiltration of macrophages, which further facilitated tumor progression.

Collectively, we proposed that within the tumor microenvironment, some early activated TNBC cells will interact with macrophages to elevate the levels of ROS, which activate the ERK1/2‐c‐Jun and NF‐κB signaling pathways to increase the expression of IL1α. When IL1α molecules are secreted out of the cells, they can bind to the receptor IL1R1 either on themselves or to neighboring late‐activated TNBC cells to activate three downstream signaling pathways: 1) ERK1/2‐ZEB1‐VIM; 2) MKK4‐JNK‐c‐Jun; and 3) NF‐κB. These pathways further increase the expression of IL1α, IL1β and IL8 to increase the survival of TNBC when they interact with macrophages, and enhance the tumor growth, metastasis of TNBC, and macrophage infiltration into tumor sites (Figure 9C).

## 3 Discussion

TNBC has a higher mortality rate than non‐TNBC, as TNBC cells are more likely to metastasize to other parts of the body. In addition to metastasis‐promoting factors in the cancer cells, macrophage infiltration into the TME can also increase cancer metastasis.^[^ ^37^ ^]^ For example, analysis of clinical tumor samples showed that TNBC tumors had more TAM infiltration than non‐TNBC tumors, which was correlated with a shorter survival rate of patients with TNBC.^[^ ^38^, ^39^ ^]^ Studies have reported that TNBC cells have a stronger ability to induce M2 macrophage differentiation and are more likely to acquire drug resistance than non‐TNBC cells.^[^ ^24^, ^40^, ^41^ ^]^ These studies focused more on macrophages, while how cancer cells become more malignant after interacting with macrophages is less reported. In this study, by using a 3D co‐culture system, we found that TNBC cells displayed stronger viability than non‐TNBC cells when co‐cultured with macrophages. More importantly, co‐culturing with macrophages greatly increased cancer cell migration, macrophage infiltration, tumor growth, and cancer metastasis.

RNA‐seq analysis revealed that IL1α, IL1β and IL8 were significantly upregulated in the co‐cultured TNBC cells and their high expression, especially IL1α and IL8, is important for TNBC cells to achieve aforementioned tumorigenic and metastatic phenotypes. In consistent with our findings, previous studies have also reported that the high expression of IL1α, IL1β or IL8 could increase tumor growth and metastasis of various types of cancer cells, including TNBC cells.^[^ ^42^, ^43^, ^44^, ^45^, ^46^, ^47^, ^48^, ^49^, ^50^, ^51^, ^52^, ^53^, ^54^, ^55^, ^56^ ^]^ Furthermore, elevating the expression of IL1β or IL8 in macrophages after the co‐culture treatment enhanced breast tumor growth and metastasis.^[^ ^57^, ^58^ ^]^

Recently, Geng et al. reported that treating macrophages with recombinant proteins of IL1α, IL8, chemokine (C‐C motif) ligand 2 (CCL2), and urokinase‐type plasminogen activator (uPA) or pancreatic cancer cells overexpressing these proteins increased macrophage invasion.^[^ ^59^ ^]^ In our study, we found that in addition to IL1α and IL8, IL1β could also increase macrophage infiltration. Moreover, our RNA‐seq results showed that the mRNA levels of CCL2 and uPA were not significantly elevated in co‐cultured 231‐GFP cells.

Although the mRNA levels of IL1α, IL1β and IL8 were all significantly upregulated after co‐culture treatment, the protein levels of the first two were significantly elevated at 48 h, while that of IL8 was greatly elevated at 72 h. More importantly, overexpression and knockdown experiments showed that IL1α controlled the expression of IL1β and IL8 in TNBC 231‐GFP cells, which indicates IL1α as a critical cytokine in macrophage‐stimulated tumor progression. Another supporting evidence for the importance of IL1α is that knockdown of its receptor (IL1R1) greatly reduced macrophage‐stimulated tumorigenesis and metastasis of TNBC. Our findings can be supported by a previous report which showed that addition of recombinant IL1α proteins to TNBC cells increased the mRNA levels of IL8. Although another study reported that addition of recombinant IL1β proteins to TNBC cells increased the mRNA levels of IL8,^[^ ^56^, ^60^ ^]^ we did not find a direct correlation between IL1β and IL8.

Other studies have previously reported that ROS can increase the expression of different cytokines, including IL1α, IL1β and IL6, in keratinocytes^[^ ^61^ ^]^ and IL1α in liver cells.^[^ ^62^ ^]^ In this study, we found that the levels of ROS were elevated within 12–16 h of co‐culture in some early activated TNBC cells. This ROS elevation is required to activate ERK1/2‐c‐Jun and NF‐κB at 20–24 h after co‐culture and to further increase the expression IL1α, IL1β and IL8 in TNBC cells. Our previous study and others’ reports also showed that increasing ROS elevation enhanced migration and invasion of cancer cells.^[^ ^35^, ^63^ ^]^ Therefore, co‐culture may increase metastasis of TNBC cells by elevating ROS levels.

It is known that IL1α can bind to its receptor IL1R1 and IL1R1 accessory protein (IL1RAP) on the cell membrane to recruit the adaptor protein of myeloid differentiation primary response 88 (MyD88) and activate IRAK4. IRAK4 can further activate the downstream signaling pathways of MAPKs/AP‐1, NF‐κB, PI3K/Akt, and JAK/STAT3.^[^ ^52^, ^55^, ^64^, ^65^, ^66^ ^]^ In this study, we found that ILα might activate its downstream signaling pathways by binding to IL1R1 in autocrine or paracrine manners to activate ERK1/2‐ZEB1‐VIM pathway to increase metastasis of TNBC cells. The secreted IL1α could also stimulate IRAK4 to activate MKK4‐JNK‐c‐Jun and NF‐κB pathways to increase the expression of IL1α, IL1β and IL8 in late‐activated TNBC cells. We did not observe the activation of PI3K/Akt and JAK/STAT3 signaling pathways when 231‐GFP cells were co‐cultured with macrophages.

This study identified IL1α as a macrophage inducible cytokine to increase the malignancy of TNBC cells and reducing IL1α production using antioxidants may be a good strategy to decrease metastasis of TNBC. Given the role of IL1R1 in tumor progression, our findings suggest that preventing the binding of IL1α and its receptor by using antibodies against IL1α or IL1R1 could be new therapeutic strategies to reduce malignancy of TNBC. Furthermore, IL1α can serve as a potential diagnostic marker for indicating more aggressive TNBC.

## 4 Experimental Section

### Cell Lines and Cell Culture

The breast cancer cell lines MDA‐MB‐231, MDA‐MB‐468, MCF7, BT474, and T47D; the lung cancer cell line A549; the cervical cancer cell line HeLa; the human monocytes THP‐1 and human embryonic kidney cell line 293T were obtained from the American Type Culture Collection (ATCC). The mouse macrophage Raw264.7 cells were obtained from Prof. Tzu‐Ming LIU of the Faculty of Health Sciences at the University of Macau, Macao, China. The M1A‐C3 cell line was isolated from the lung metastatic tumors of female athymic nude mice injected with MDA‐MB‐231‐C3 cells through the tail vein twice as previously described.^[^ ^33^, ^67^ ^]^ The MDA‐MB‐231, M1A‐C3, MDA‐MB‐468, MCF7, BT474, A549, HeLa, 293T, and Raw264.7 cells were cultured in a Dulbecco's modified Eagle's medium (DMEM; #12100046, Thermo Fisher Scientific, USA). The T47D and THP‐1 cells were cultured in an RPMI 1640 medium (#2174257, Thermo Fisher Scientific, USA). The THP‐1 monocytes were stimulated with PMA (100 ng mL^−1^) to differentiate into macrophages. All the culture medium was supplemented with 10% fetal bovine serum (FBS) (#10270‐106, Gibco, USA) and 100 U mL^−1^ penicillin‐streptomycin (#15140122, Thermo Fisher Scientific, USA). The cells were cultured at 37 °C in a humidified incubator with 5% CO_2_. M1A‐C3, MCF7‐C3, A549‐C3, and the HeLa‐C3 cells were generated by transfecting a sensor C3 plasmid that encodes a FRET‐based biosensor named C3. 231‐GFP, BT474‐clover, and the T47D‐clover cells were transfected with green fluorescent protein GFP or clover. The Raw264.7‐tdT and THP1‐tdT cells were transfected with the red fluorescent protein tandem dimer Tomato (tdT). All cell lines used in this study were tested for Mycoplasma by the core facility of Faculty of Health Sciences of University of Macau to ensure they are not contaminated with Mycoplasma.

### Tumor Sphere Formation Assay

Green‐colored cancer cells and red‐colored macrophages were seeded in 96‐well ultralow adherent round bottom plates (#7007, Corning, USA) at a ratio of 30:1. The formed tumor spheres were imaged daily by using a Carl Zeiss microscope for seven days. ImageJ software was used to analyze the green fluorescence intensity to indicate the viability of cancer cells.

### Co‐Culture Experiment

For the physical contact co‐culture, the cancer cells and macrophages were seeded into culture dishes at a ratio of 30:1 and co‐cultured for the indicated period. Afterward, less‐attached macrophages were gently blown off from the well‐attached cancer cells by using a pipette. The washed‐off macrophages were collected from the culture medium, while the attached cancer cells were trypsinized and collected independently. For the separated co‐culture, the cancer cells and macrophages were seeded into the lower and upper chambers of a six‐well Transwell insert with 0.4 µm pores (#3450, Corning, USA), respectively.

### Transwell Migration and Invasion assay

The cell migration and invasion assays were performed in a Transwell chamber with 8 µm pores (# 3422, Corning, USA). Briefly, the breast cancer cells (1 × 10^4^) or macrophages (1 × 10^4^) in 100 µL of the serum‐free medium were added to the upper chamber, and 600 µL of the medium with 10% FBS was added to the lower chamber. The cells migrated from the upper side to the lower side of the chamber for 18 h of incubation for the cancer cells and 24 h of incubation for the macrophages. After that, the cells that remained on the top side of the Transwell membrane were gently removed using a cotton swab, while the cells on the bottom side were fixed with 4% paraformaldehyde (PFA; #158127, Sigma‐Aldrich, Germany) for 20 min and then stained with 0.1% crystal violet (#C6158, Sigma‐Aldrich, Germany) for 15 min. The membrane was then cut off and fixed on a glass slide with a mounting medium (#06522, Sigma‐Aldrich, Germany). The migrated cells were imaged with bright‐field microscopy (M165 FC, Leica, Germany).

For the invasion assay, the upper chamber was precoated with 100 µL of Matrigel (#356230, Corning, USA) at 37 °C for 2 to 3 h before the cells were seeded. The following steps were the same as those described for the migration assay.

### Immunofluorescence Staining

The cells were seeded on coverslips in a six‐well plate, washed once with PBS, fixed with 4% PFA for 20 min, and permeabilized with 0.2% Triton X‐100 (#T8787, Sigma‐Aldrich, Germany) for 20 min. After blocking with 3% bovine serum albumin (BSA) for 1 h at room temperature, the cells were incubated with a primary antibody at a 1:100 dilution overnight at 4 °C, followed by staining with the Alexa Fluor–conjugated secondary antibody at a 1:100 dilution for 1 h at room temperature. After the nuclei were stained with Hoechst 33342 (#H3570, Thermo Fisher Scientific, USA), the coverslip was mounted onto a clean glass microscope slide using Mowiol 4–88 (#475904, Calbiochem, Merck). Images were acquired using a confocal laser‐scanning microscope (Carl Zeiss LSM710, Germany).

### Matrigel Plug Assay

The serum‐free conditioned medium from the different cell lines was precooled and mixed with Matrigel at a ratio of 1:1. A volume of 200 µl of the conditioned medium was subcutaneously injected into BALB/c athymic nude mice. Matrigel plugs were maintained for 10 days in the mice and then fixed with 4% PFA immediately followed by embedding in paraffin with a Leica EG1150 embedding center. Five‐micron sections were prepared with a Leica RM2235 microtome, deparaffinized using a Leica Multistainer, and subjected to antigen retrieval using 0.1 m sodium citrate solution. The sectioned Matrigel plugs were immunofluorescence stained with antibodies of macrophage markers F4/80, CD204, and CD206. The fluorescence images were obtained by using a confocal laser‐scanning microscope (Carl Zeiss LSM710, Germany).

### RNA Sequencing Analysis

The 231‐GFP cells were monocultured or co‐cultured with Raw264.7‐tdT macrophages for 96 h, and then the collected 231‐GFP cells were lysed with TRIzol reagent (#15596026, Invitrogen, USA). The RNA sequencing was performed by Novogene Company (Tianjin, China) for RNA extraction (RNA integrity number > 9), purification, library preparation, sequencing, and basic data analysis. A false discovery rate (FDR) < 0.05 and a |log2 (Fold change) | ≥ 1 were used as the thresholds to identify the differentially expressed genes with statistical significance.

### Real‐Time Quantitative Polymerase Chain Reaction (qPCR)

Total RNA was extracted with TRIzol reagent, and reverse transcription reactions were subsequently performed with the iScriptTM cDNA Synthesis Kit (#1778890, Bio‐Rad, USA) according to the manufacturer's protocol. The real‐time PCR was performed in triplicate using the iTaqTM Universal SYBR Green Supermix (#1725122, Bio‐Rad, USA) on the CFX96 TouchTM Real‐Time PCR Detection System (Bio‐Rad, USA). Relative quantification was performed using the ∆∆CT method. GAPDH served as the internal control. The species‐specific primers used for real‐time PCR were listed in Table S1 (Supporting Information).

### Lentivirus‐Delivered shRNA Knockdown and Gene Overexpression

All the shRNA and overexpression vectors were purchased from the VectorBuilder Company (Chicago, USA), and the target sequences of the genes in this study are listed in Tables S2,S3 (Supporting Information). The selected shRNA sequences were cloned into the pLKO.1‐puro lentiviral construct. Recombinant lentiviral particles were produced by transient transfection of plasmids into the 293T cells. In brief, 0.25 µg of pMD2. G (encoding the VSVG envelope protein), 0.5 µg of pCMVR8.2 (encoding HIV‐1 Gag, Pol, Tat, and Rev proteins), and 0.75 µg of gene‐targeted plasmids were transfected using polyethylenimine linear (PEI) (#23966, polysciences, USA) into the 293T cells at a density of 1 × 10^6^ cells in 6‐well plates. The viral supernatant was collected at 36 h and 72 h after transfection and filtered with a 0.45 µm filter. The viral particles were used to infect host cells, while the positive cells were selected with 2 µg mL^−1^ puromycin (#P8833, Sigma, Germany) or 10 µg mL^−1^ blasticidin (#ant‐bl‐05, InvivoGene, USA). The knockdown or overexpression efficiency in the targeted cells was tested by using qPCR or Western blotting.

### Western Blotting

The samples were collected and lysed in radioimmunoprecipitation assay buffer (RIPA; 150 mM NaCl, 50 mM Tris‐HCl, 0.5% SDS, and 1% Triton X‐100) supplemented with a protease inhibitor (#P8340, Sigma‒Aldrich, Germany) and phosphatase inhibitor cocktail 2 and 3 (#P0044, #P5726, Sigma‒Aldrich, Germany) on ice for 30 min. After sonication and centrifugation, the total protein concentration was determined by the Bio‐Rad protein assay using Dye Reagent Concentrate (#5000006, Bio‐Rad, USA). Equal amounts of protein from each treatment group were separated by SDS‐PAGE and electro‐transferred to a nitrocellulose membrane (#1620112, Bio‐Rad, USA). The membrane was probed with a specific primary antibody followed by incubation with the HRP‐conjugated secondary antibody. Finally, immunoreactivity was detected by the Chemiluminescent Substrate (Clarity Western ECL Substrate; Bio‐Rad, USA). The primary and secondary antibodies used in this study are listed in Table S4 (Supporting Information).

### Intracellular ROS Detection

The MDA‐MB‐231 cells and Raw264.7 macrophages were co‐cultured in a Transwell chamber for the duration of different time points. The MDA‐MB‐231 cells were then immediately stained with 10 µM CM‐H2DCFDA (DCFDA; #C6827, Thermo Fisher Scientific, USA) in serum‐free fluoro‐brite medium (#A1896701, Thermo Fisher Scientific, USA) for 30 min at 37 °C. The images were taken with a Carl Zeiss microscope and analyzed by using ImageJ software.

### Lung Colony Formation Assay

All the mice were abstained and maintained in the Animal Facility of the University of Macau, and all animal experiments were approved by The Animal Research Ethics Committee of the University of Macau (Approved Protocol ID: UMARE‐025‐2017 and UMARE‐026‐2017). After the co‐culture treatment, 2.5 × 10^6^ breast cancer cells with green fluorescence and 2.5 × 10^6^ macrophages with red fluorescence were mixed with 100 µl of PBS and intravenously injected into 6‐ to 8‐week‐old female BALB/c athymic nude mice. Before injection, mice were randomly divided into different groups with sufficient number of animals per group to ensure that at least there were 6 mice remaining at the end of experiments. This sample size was designed by power analysis with a level of significance of 0.05. After 28 days, the mice were sacrificed, and the harvested lung tissues were washed once with PBS. The lung tissues were placed on a 6‐cm petri dish and the fluorescence images were taken using a fluorescence dissecting microscope (Leica Microsystems Ltd. M165 FC, Germany or Olympus MVX10, Japan).

### Primary Tumor Growth and Spontaneous Metastasis in an Orthotopic Mouse Model

Female NOD/SCID mice (6–8‐week‐old) were randomly divided into different groups. TNBC (231‐GFP) cells and human macrophages (THP1‐tdT) were co‐cultured for 96 h, afterward, 2 × 10^6^ 231‐GFP cells and 4 × 10^5^ macrophages were mixed and injected into the fourth pair of mammary fat pads. The injected cells were allowed to grow into primary xenograft breast tumors and metastasize to different organs for 42 days. During this period, the tumor size and body weight were measured twice a week. The tumor volume was calculated using the following formula: length × (width)^2^/2. At the end of animal experiment, mice were sacrificed, and the weights of xenograft tumors were measured. The iliac lymph nodes and the lung metastatic tumors were imaged by fluorescence microscopy. If the mice died during the experiments, the animal data were excluded. To ensure that at least six mice could remain at the end of animal experiments, sufficient number of mice were used at the beginning of these experiments and this sample size could also meet the required power analysis with a level of significance of 0.05.

### Immunohistochemistry

Tissue microarray slides of TNBC patients were purchased from Shanghai Outdo Biotech Company (Shanghai, China). Experiments on the human tissue microarray were approved by the Ethics Committee of Shanghai Outdo Biotech Company (Approval No.: SHXC2021YF01). IHC was performed using the IHC Detection Kit (#ab64264, Abcam, UK) according to the manufacturer's instructions. The tumor xenograft tissues were fixed with 4% PFA, embedded in paraffin after dehydration, and subsequently sectioned to a thickness of 5 µm. The paraffin‐embedded tissue sections were deparaffinized, rehydrated, and boiled with a 0.1 m sodium citrate buffer for 20 min to retrieve the antigens. The endogenous peroxidase activity was blocked by using hydrogen peroxide, and nonspecific binding was prevented by using a protein‐blocking buffer. Slides were then incubated with primary antibodies at 4 °C overnight. The sections were incubated with biotinylated secondary antibody for 10 min. The antigen‐antibody interaction was revealed with the streptavidin‐biotin–horseradish peroxidase system using diaminobenzidine as a chromogen. Nuclei were counterstained with hematoxylin (#H3136, Merck, Germany). Images were acquired at 20× magnification using an Aperio scanner system (Leica, Germany). The expression levels of the target proteins were determined by semiquantitative analysis. IHC scores were calculated based on the percentage of stained cells.^[^ ^68^, ^69^ ^]^ The percentage scores were assigned as follows: 0, none; 1, <1% of positively stained cells; 2, 1 to 10%; 3, 11 to 33%; 4, 34 to 66%; and 5, 67 to 100%.

### Statistical Analysis

All the results were obtained from three independent experiments or from six mice. Data are presented as the mean ± standard deviation (SD). Student's t‐test, one‐way analysis of variance (ANOVA), or two‐way ANOVA was used for statistically significant analysis using GraphPad 9.0. Significance was indicated as follows: *p < 0.05, **p < 0.01, ***p < 0.001, and ****p < 0.0001.

## Conflict of interest

The authors declare no conflict of interest.

## Author Contributions

M.H. and K.Q.L. conceived the study and designed the experiments. M.H. conducted the experiments. B.H., R.W., and Z.P. helped with some of the experiments. M.H. and K.Q.L. analyzed the data and wrote the manuscript.

## Supporting information

Supporting Information

## Data Availability

The data that support the findings of this study are available from the corresponding author upon reasonable request.
