# Supplementary material for: The Interaction between Macrophages and Triple‐negative Breast Cancer Cells Induces ROS‐Mediated Interleukin 1α Expression to Enhance Tumorigenesis and Metastasis
Source: Adv Sci (Weinh). 2023 Aug 8;10(29):2302857. doi: 10.1002/advs.202302857 (PMC10582438; doi:10.1002/advs.202302857)
Supplement: Supplementary file 1 — Supporting Information [file ADVS-10-2302857-s001.pdf]

ADVANCED SCIENCE
Open Access

Supporting Information

for *Adv. Sci.*, DOI 10.1002/advs.202302857

The Interaction between Macrophages and Triple-negative Breast Cancer Cells Induces ROS-Mediated Interleukin 1α Expression to Enhance Tumorigenesis and Metastasis

*Meng Hao, Bin Huang, Renfei Wu, Zheng Peng and Kathy Qian Luo**

Supporting Information

The Interaction between Macrophages and Triple-negative Breast Cancer Cells Induces ROS-mediated Interleukin 1α Expression to Enhance Tumorigenesis and Metastasis

Meng Hao[1], Bin Huang[1], Renfei Wu[1], Zheng Peng[1], Kathy Qian Luo[1, 2]*

Supplementary figures

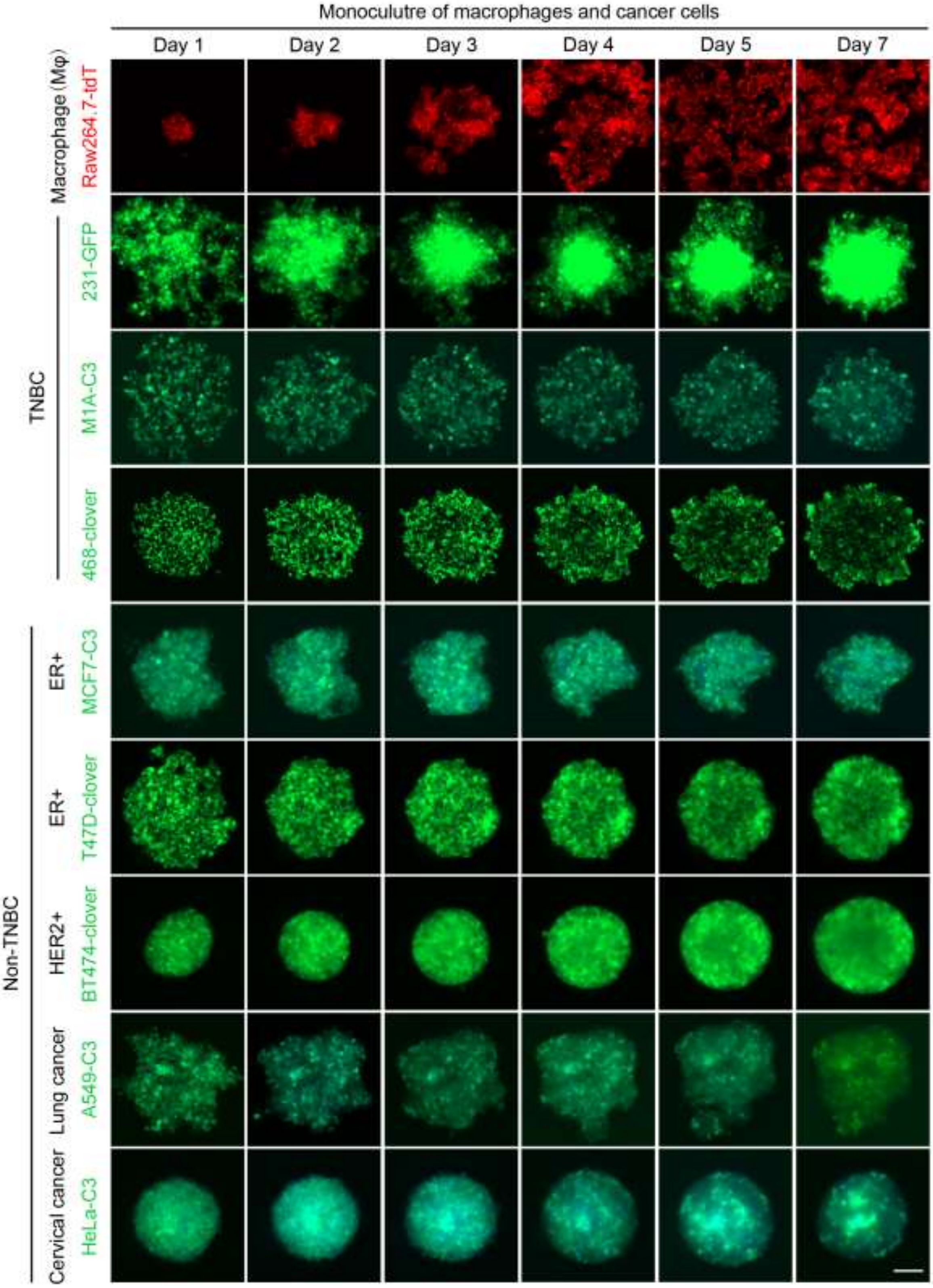

Figure S1. Macrophages and cancer cells were monocultured in 3D conditions. Macrophages and multiple types of cancer cells were monocultured in the low-attachment round bottom 96-well plates for 1–7 days. The number of macrophages at the initial of 3D

culture was 67 and the number of cancer cells at initial was 2000. tdT: red fluorescent protein; GFP and clover: green fluorescent protein; C3: apoptotic sensor cells emit green fluorescence in live cells and emit blue fluorescence in apoptotic cells.

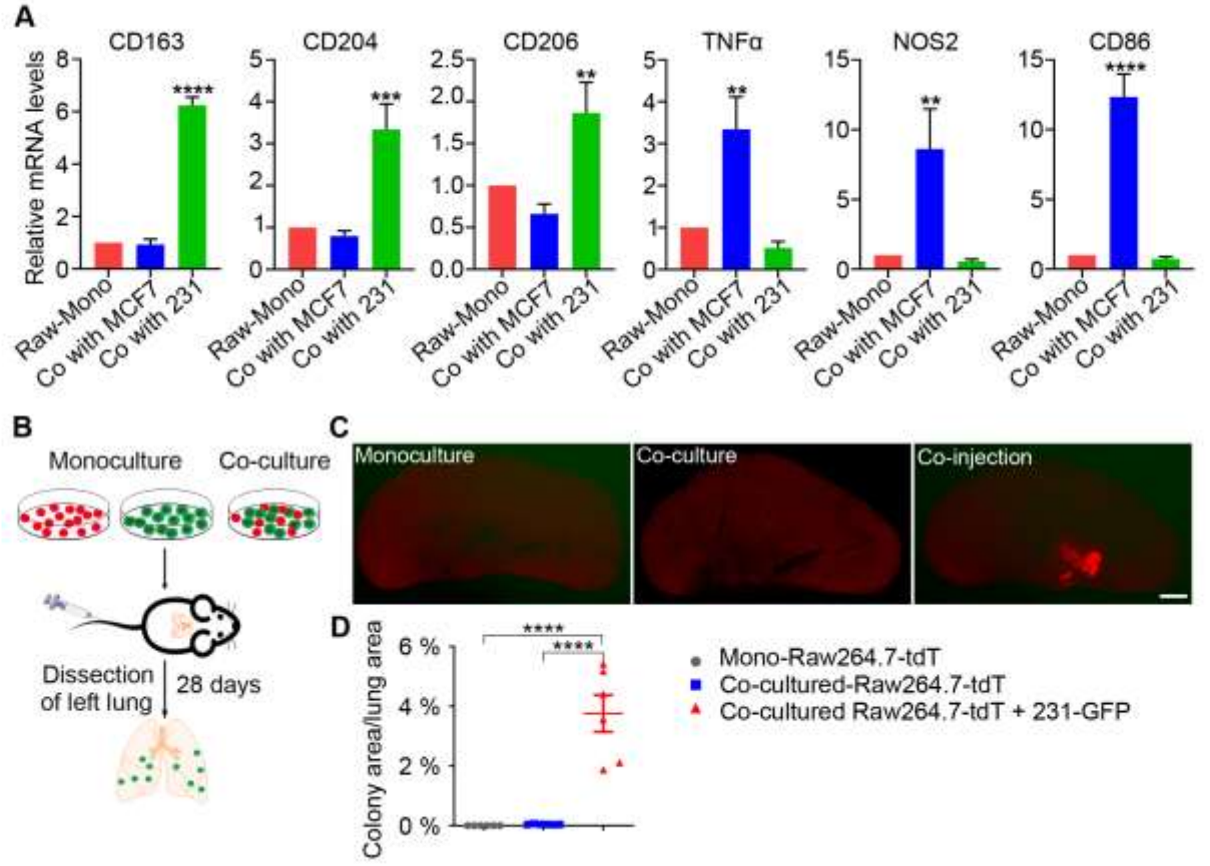

Figure S2. Macrophages injected alone into the nude mice failed to form any lung colonies and TNBC cells induced macrophage polarization to M2 type. (**A**) qPCR results showing the mRNA levels of M1 macrophage markers TNFα, NOS2, CD86 and M2 macrophage markers of CD163, CD204 and CD206 in Raw264.7 cells before and after 96 h of co-culturing with 231-GFP or MCF-7 cells. The results represent the means ± SD from three independent experiments or form six mice. (**B)** Schematic diagram of the tail vein injection with mono- or co-cultured cells. (**C**) Representative lung images from the mice injected with mono- or co-cultured Raw264.7-tdT macrophages alone or Raw264.7-tdT co-cultured with 231-GFP cells through the tail vein for 28 days. (n = 6 mice per group). Scale bar: 1 mm. (**D**) Percentage of the colony area formed by macrophages to the total left lung area of each treatment group. Significant differences were determined by one-way ANOVA. $^{**}P < 0.01$, $^{***}P < 0.001$, $^{****}P < 0.0001$.

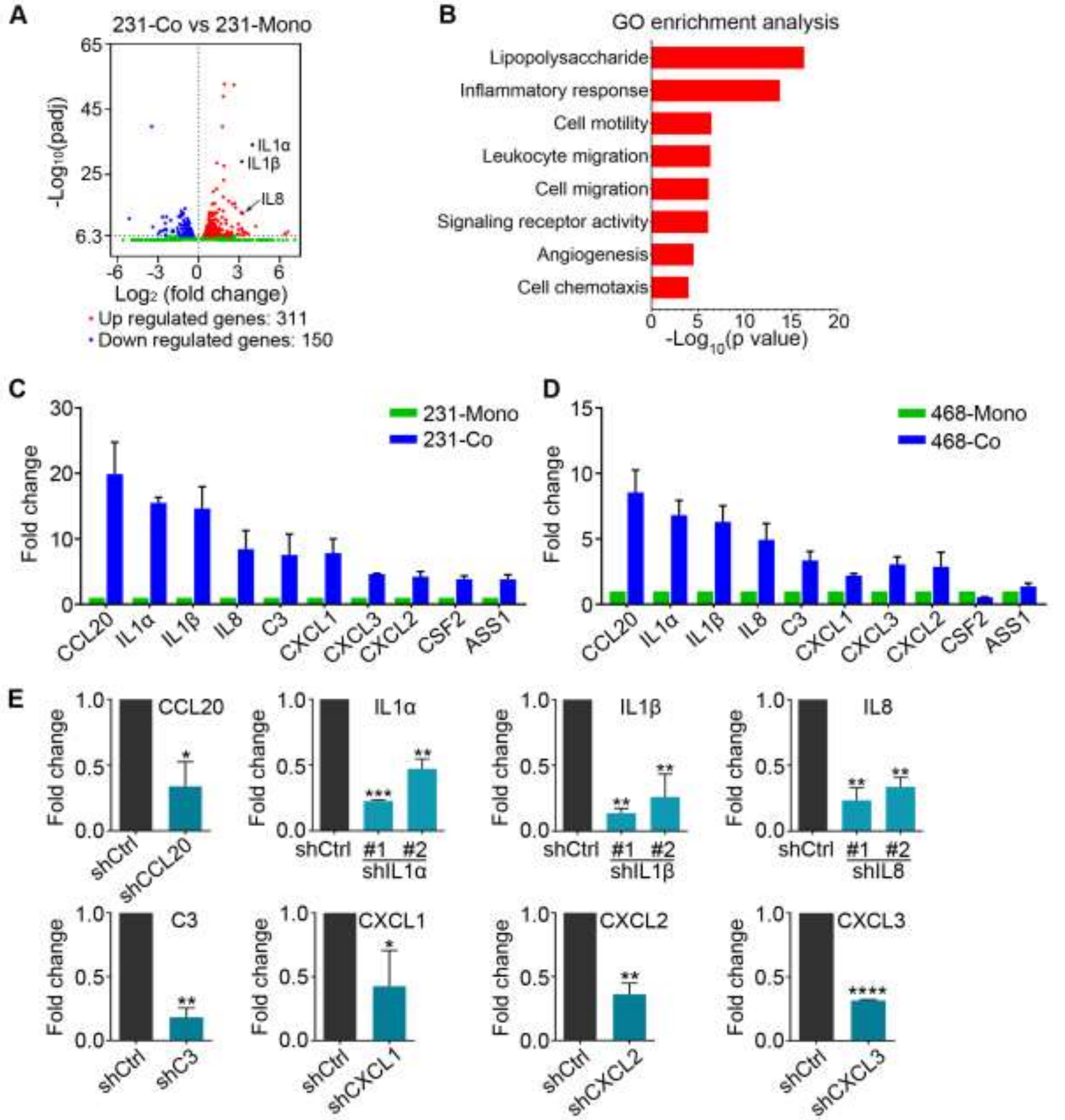

Figure S3. Identification of the driver genes that were upregulated in the co-cultured TNBC cells. (**A**) Volcano diagram of the differentially expressed genes. Red dots indicate upregulated genes, and blue dots indicate downregulated genes with the cut-off criteria of fold change > 1 and P < 0.05. (**B**) GO enrichment analysis of the upregulated signaling pathways. (**C** and **D**) qPCR validation of the top 10 upregulated secreted genes in the 231-GFP or MDA-MB-468 cells after being co-cultured with Raw264.7-tdT macrophages. (**E**) qPCR results of the knockdown efficiency of the target genes in the 231-GFP cells with shRNAs. The results represent the means ± SD from three independent experiments. Significant differences were determined by Student's t-test. **P* < 0.05, ***P* < 0.01, ****P* < 0.001 and *****P* < 0.0001.

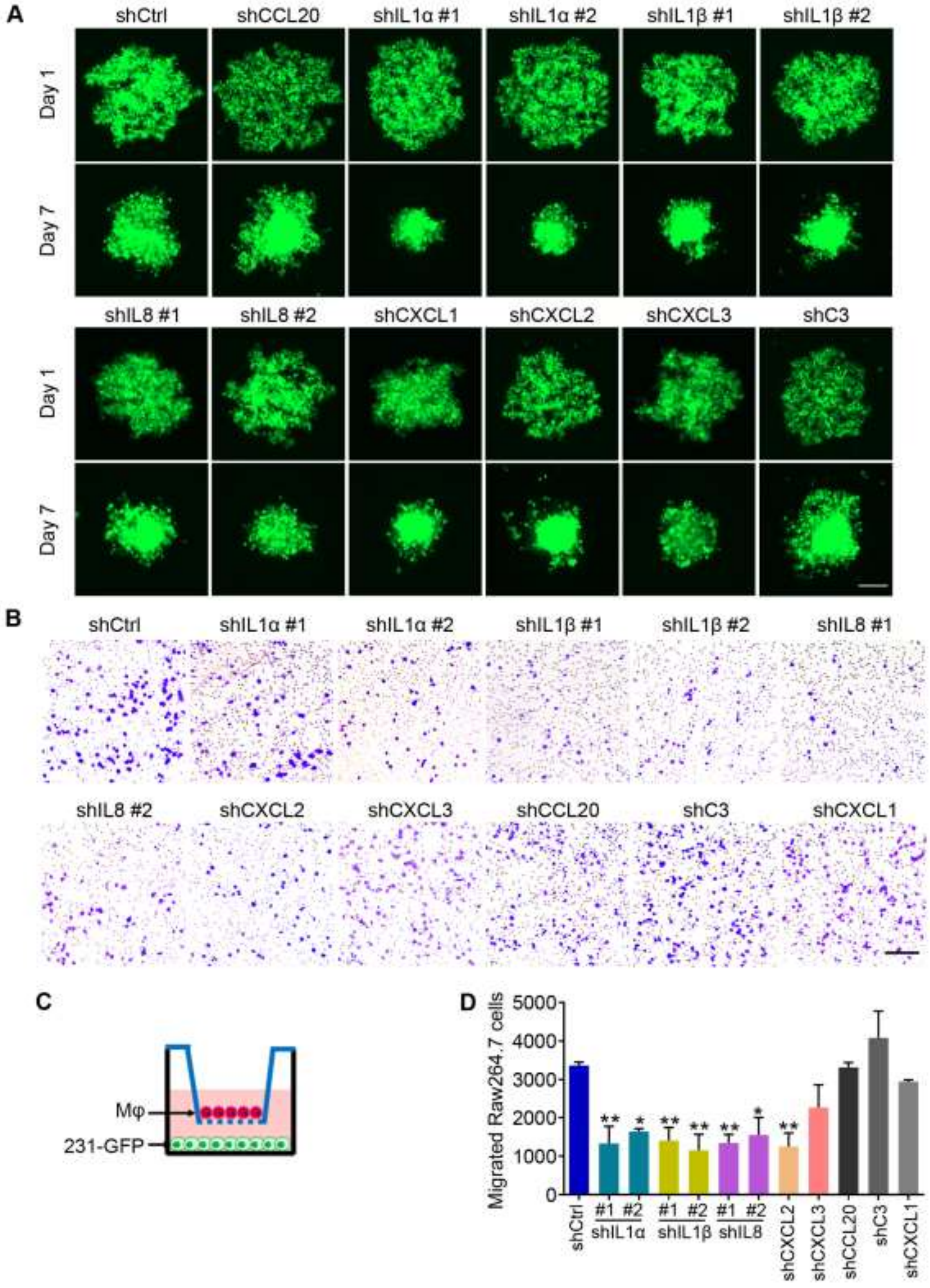

Figure S4. Determining the importance of the candidate genes in enhancing the viability of 231-GFP cells and migration ability of Raw264.7 macrophages. (**A**) Representative fluorescence images of the tumor spheres formed by the monocultured 231-GFP cells with or without the target gene knockdown. Scale bar, 200 μm. (**B and C**) Schematic diagram (C) and representative images (B) of the Transwell analysis of the Raw264.7 macrophages with the

231-GFP cells seeded in the lower chamber of the Transwell insert. Scale bar, 200 μm. (**D**) The quantified number of the migrated Raw264.7 cells. The results represent the means ± SD from three independent experiments. Significant differences were determined by one-way ANOVA. *P < 0.05 and **P < 0.01.

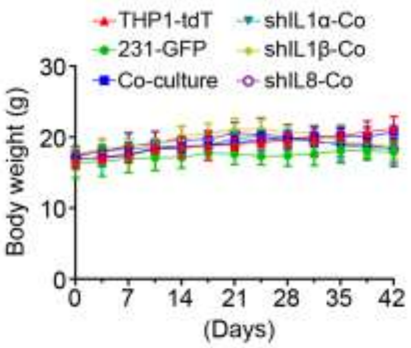

Figure S5. Body weights of NOD/SCID mice implanted with orthotopic tumors. Body weight of the NOD/SCID mice inoculated with mono- or co-cultured THP1-tdT macrophages and 231-GFP cells with or without the knockdown of IL1α, IL1β and IL8. (n = 6 NOD/SCID mice per group).

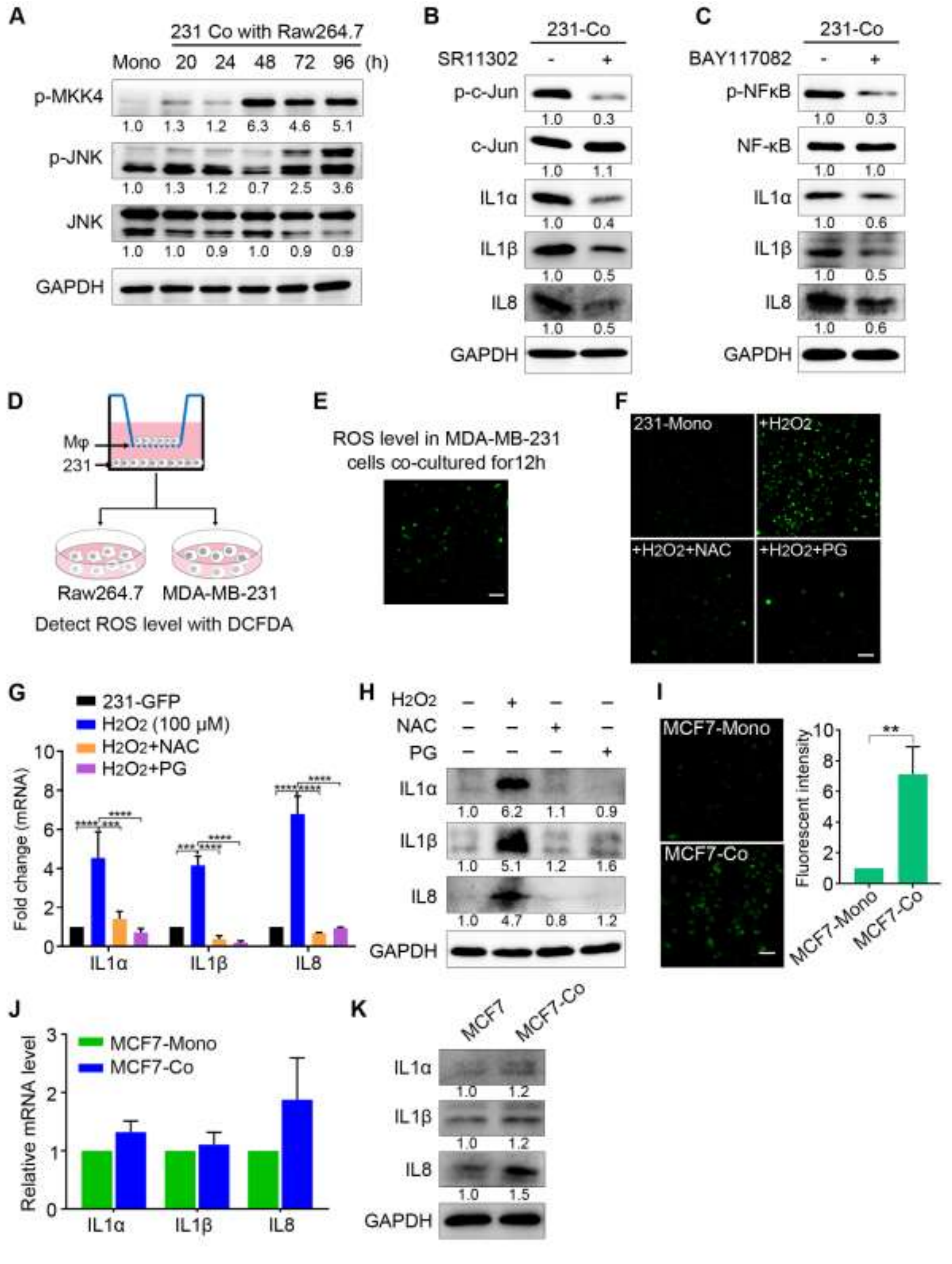

Figure S6. ROS elevation activated the ERK1/2-c-Jun and NF-κB signaling pathways to upregulate the expression of IL1α, IL1β and IL8 in TNBC cells. (**A**) Western blotting of the protein levels of p-MKK4, JNK and p-JNK in the mono- or co-cultured 231-GFP cells. (**B**) Western blotting of c-Jun, p-c-Jun, IL1α, IL1β and IL8 in the 231-GFP cells with or without the treatment of the c-Jun inhibitor SR11303 (5 μM) during 96 h of the co-culture treatment

with Raw264.7 cells. The medium containing the inhibitor was replaced every two days. (**C**) Western blotting of NF-κB, p-NF-κB, IL1α, IL1β and IL8 in 231-GFP cells with or without the treatment of the NF-κB inhibitor BAY117082 (2.5 μM) during 96 h of the co-culture treatment with Raw264.7 cells. The medium containing the inhibitor was replaced every two days. (**D**) Schematic diagram showing the ROS detection in the MDA-MB-231 cells with DCFDA dye after the separated co-culture treatment with Raw264.7 macrophages. (**E**) DCFDA-stained fluorescence images of MDA-MB-231 cells co-cultured for 12 h. Scale bar, 100 μm. (**F**) DCFDA-stained fluorescence images of the MDA-MB-231 cells, with or without the treatment with H_2O_2 (100 μM), NAC (5 mM) and PG (20 μM). Scale bar, 100 μm. (**G** and **H**) qPCR and Western blots showing the mRNA and protein levels of IL1α, IL1β and IL8 in 231-GFP cells with or without the treatment with H_2O_2 (100 μM), NAC (5 mM) and PG (20 μM). (**I**) (left) DCFDA-stained fluorescence images of the mono- or 16 h co-cultured MCF-7 cells. Scale bar, 100 μm. (right) Quantified fluorescence intensity of ROS in MCF-7 cells. (**J** and **K**) qPCR and Western blots showing the mRNA and protein levels of IL1α, IL1β and IL8 in MCF-7 cells after they were co-cultured with Raw264.7 macrophages. The results represent the means ± SD from three independent experiments. Significant differences were determined by one-way ANOVA (G) and Student's t-test (I). ***P* < 0.01, ****P* < 0.001 and *****P* < 0.0001.

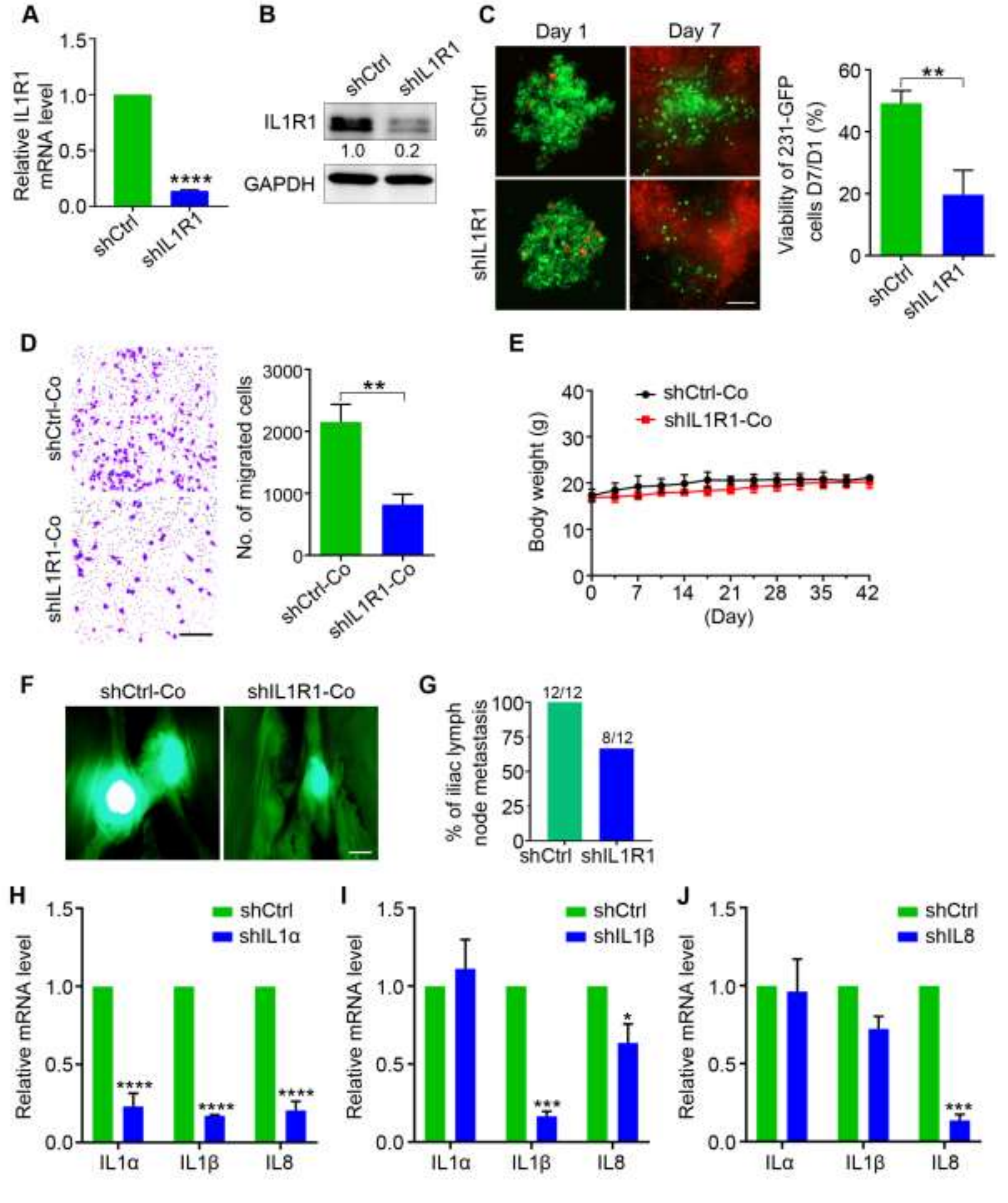

Figure S7. Knockdown of IL1R1 reduced the viability and migration of the 231-GFP cells. (**A** and **B**) qPCR and Western blots showing the mRNA and protein levels of ILR1 in the 231-GFP cells transfected with IL1R1 shRNA. (**C**) (left) Representative fluorescence images of the tumor spheres formed by the 231-GFP cells with or without the IL1R1 knockdown when co-cultured with Raw264.7-tdT cells. Scale bar, 200 μm. (right) Quantification of the total green fluorescence intensity indicated the viability of the 231-GFP cells on day 7 compared to day 1. (**D**) Representative images and the quantified results of the Transwell migration assay of the 231-GFP cells with or without the IL1R1 knockdown. Scale bar, 200 μm. (**E**) Body weight of the NOD/SCID mice inoculated with co-cultured THP1-tdT macrophages plus 231-GFP cells

with or without the IL1R1 knockdown. (**F**) Representative images of the iliac lymph node metastatic tumors of each treatment group which were harvested on day 42. Scale bar, 1 mm. (**G**) Incidence of lymphatic metastasis of each treatment group. (**H** to **J**) qPCR results showing the mRNA levels of IL1α, IL1β and IL8 when each of them was knocked down in the 231-GFP cells. The results represent the means ± SD from three independent experiments or from six mice. Significant differences were determined by Student's t-test. *$P < 0.05$, **$P < 0.01$, ***$P < 0.001$ and ****$P < 0.0001$.

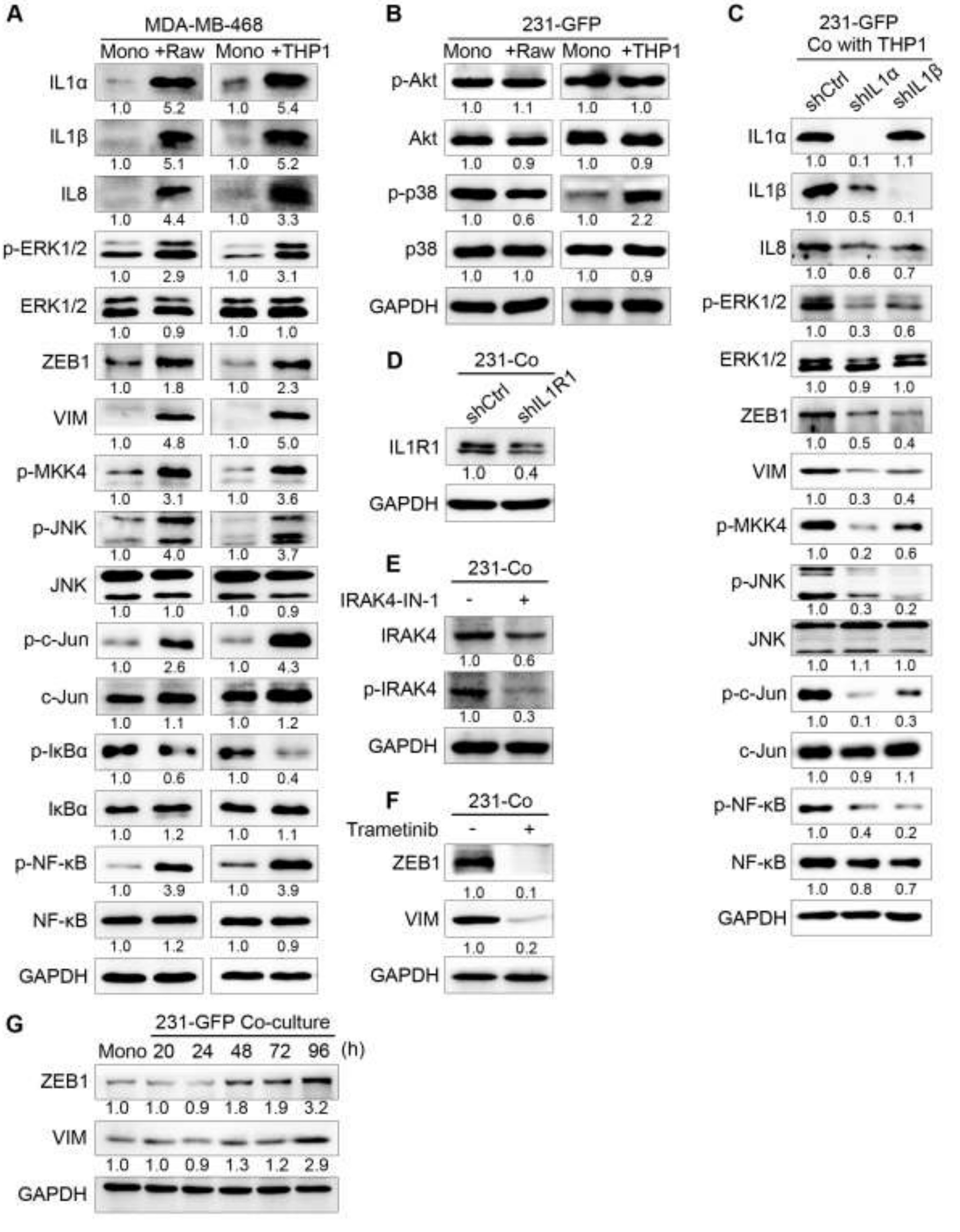

Figure S8. IL1α activated three signaling pathways mediated by ERK1/2, MKK4 and NF-κB during the co-culture of TNBC cells with macrophages. (**A**) Western blots showing the total or phosphorylated protein levels of IL1α, IL1β, IL8, ERK1/2, ZEB1, VIM, MKK4, JNK, c-Jun, IκBα and NF-κB in the MDA-MB-468 cells before and after 96 h of the co-culture treatment with Raw264.7-tdT or THP1-tdT macrophages. (**B**) Western blots showing the total

or phosphorylated Akt and p38 in the 231-GFP cells before or after 96 h of the co-culture treatment with Raw264.7-tdT or THP1-tdT macrophages. (**C**) Western blots showing the knockdown effects of IL1α and IL1β on themselves and other indicated proteins in the 231-GFP cells co-cultured with THP1-tdT macrophages for 96 h. (**D**) Western blots showing the expression level of IL1R1 in the co-cultured 231-GFP cells with or without the IL1R1 knockdown. (**E**) Western blotting of the IRAK4 and p-IRAK4 in co-cultured 231-GFP cells with or without the addition of IRAK4 inhibitor (IRAK4-IN-1, 5 μM) during the co-culture treatment. (**F**) Western blotting of ZEB1 and Vimentin in the 231-GFP cells with or without the addition of MEK inhibitor (trametinib, 10 nM) during the co-culture treatment with Raw264.7-tdT cells. (**G**) Western blotting of ZEB1 and Vimentin in the mono- or co-cultured 231-GFP cells.

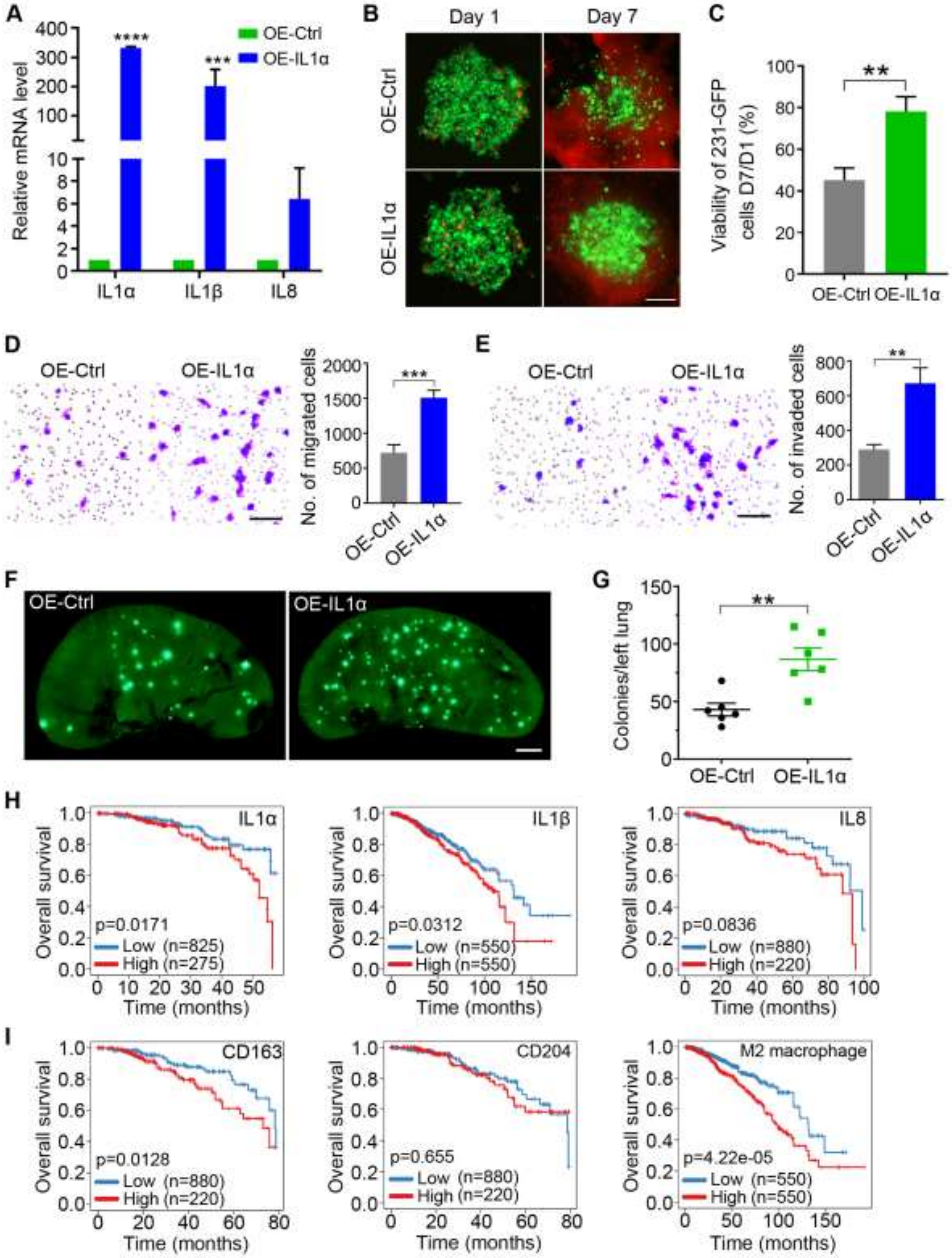

Figure S9. Overexpression of IL1α upregulated IL1β and IL8 and promoted the survival, migration, invasion and lung metastatic ability of 231-GFP cells. (**A**) qPCR results showing the mRNA levels of IL1α, IL1β and IL8 in the 231-GFP cells with or without the overexpression of IL1α. (**B**) Representative fluorescence images of the tumor spheres formed by the 231-GFP cells with or without the overexpression of IL1α when co-cultured with

Raw264.7-tdT cells. Scale bar, 200 μm. (**C**) Quantification of the total green fluorescence intensity indicating the viability of the 231-GFP cells on day 7 compared to day 1. (**D** and **E**) Representative images and the quantified results of the Transwell migration (D) and invasion (E) assays of the 231-GFP cells with or without the overexpression of IL1α. Scale bar, 200 μm. (**F** and **G**) Representative fluorescence images and the quantified lung colony number of the 231-GFP cells with or without the overexpression of IL1α on day 28 after the tail vein injection of the nude mice ($n = 6$ nude mice per group). Scale bar, 1 mm. (**H** and **I**) Correlation between the expression levels of IL1α, IL1β, IL8, CD163, CD204 or M2 macrophage infiltration and overall survival curves in patients with breast cancer analyzed on the TIMER 2.0 platform. The results represent the means ± SD from three independent experiments or from six mice. Significant differences were determined by Student's t-test. $^{**}P < 0.01$, $^{***}P < 0.001$ and $^{****}P < 0.0001$.

Supplementary tables

Table S1. List of homo specific primers.

**Gene name**	**Forward (5'-3')**	**Reverse (5'-3')**
GAPDH	CCCCACCACACTGAATCTCC	GTACATGACAAGGTGCGGCT
IL1α	TGGTAGTAGCAACCAACGGGA	ACTTTGATTGAGGGCGTCATTC
IL1β	ATGATGGCTTATTACAGTGGCAA	GTCGGAGATTCGTAGCTGGA
IL8	ACTGAGAGTGATTGAGAGTGGAC	AACCCTCTGCACCCAGTTTTC
CXCL1	ACTCTACCTGCACACTGTCCTA	CATTTGCTTGGATCCGCCAG
CXCL2	TGAATCTACTTGCACACTCTCCC	TACATTTCCCTGCCGTCACAT
CXCL3	TTCTAGGGACAGCTGGAAAGG	TCCCCACCCTGTCATTTATCA
CCL20	TGCTGTACCAAGAGTTTGCTC	CGCACACAGACAACTTTTTCTTT
C3	GGGGAGTCCCATGTACTCTATC	GGAAGTCGTGGACAGTAACAG
CSF2	GGAGCATGTGAATGCCATCCAG	CTGGAGGTCAAACATTTCTGAGAT
ASS1	TGTGCTTATAACCTGGGATGGG	CGGCGTCTGGAATCTGTTTT
IL1R1	ATGAAATTGATGTTCGTCCCTGT	ACCACGCAATAGTAATGTCCTG

Table S2. List of shRNAs.

**shRNA name**	**Target sequence (5'-3')**
shIL1α#1	TGAAACCTCTAAAACATCCAA
shIL1α#2	GCCCTCAATCAAAGTATAATT
shIL1β#1	AGCAACCGCTTCCCTATTTAT
shIL1β#2	TCACCTCTCCTACTCACTTAA
shIL8#1	CCAGATGCAATACAAGATTCC
shIL8#2	TGCGCCAACACAGAAATTATT

shIL1R1	TGGTATAGATGCAGCATATAT
shCXCL1	ATATTTTAGGTGTAAAATAAT
shCXCL2	CTTGCACACTCTCCCATTATA
shCXCL3	TTACGAGGGTTCTACTTATTT
shCCL20	TCCAAAACAGACTTGGGTGAA
shC3	AGTTCAAGTCAGAAAAGGGGG

Table S3. List of overexpression vector.

**Vector name**	**Transcript ID**	**Sequence length**
IL1α	NM_000575.5	816 bp

Table S4. List of primary and secondary antibodies.

**Antibody name**	**Company**	**Catalog #**	**Application and concentration**
IL1α, mouse	Santa Cruz	sc-9983	WB (1:500), IF (1:100), IHC (1:100)
IL1β, mouse	Santa Cruz	sc-32294	WB (1:500), IF (1:100)
IL8, rabbit	CST*	94407	WB (1:1000), IF (1:100)
IL1R1, mouse	Santa Cruz	sc-393998	WB (1:1000)
IRAK4, rabbit	CST	4363S	WB (1:1000)
p-IRAK4, rabbit	CST	11927S	WB (1:1000)
p-MKK4 (Ser257), rabbit	CST	4514	WB (1:1000)
JNK, rabbit	CST	9252	WB (1:1000)
p-JNK (Thr183/Tyr185), rabbit	CST	9251	WB (1:1000)
c-Jun, rabbit	CST	9165	WB (1:1000)
p-c-Jun (Ser73)	CST	3270	WB (1:1000)
ERK1/2, rabbit	CST	4695	WB (1: 1000)
p-ERK1/2 (Thr202/Tyr204), rabbit	CST	4370	WB (1: 1000)
ZEB1, rabbit	Novus	NBP1-88845	WB (1:1000)
Vimentin, rabbit	CST	5741	WB (1:1000)
IκBα, mouse	CST	4814	WB (1:1000)
p-IκBα (Ser32/36), mouse	CST	9246	WB (1:1000)
NF-κB, rabbit	CST	8242	WB (1:1000)
p-NF-κB (Ser536), rabbit	CST	3033	WB (1:1000)
AKT, rabbit	CST	4691	WB (1:1000)
p-AKT (Ser473), rabbit	CST	4060	WB (1:1000)

p38, rabbit	CST	8690	WB (1:1000)
p-p38 (Thr180/Tyr182), rabbit	CST	4511	WB (1:1000)
GAPDH, rabbit	CST	2118	WB (1:5000)
F4/80, rat	Abcam	ab6640	IF (1:100)
CD204, rabbit	Thermo	PA5-22596	IF (1:100), IHC (1:500)
CD206, goat	R&D	AF2535	IF (1:100)
CD163, rabbit	Abcam	Ab18442	IF (1:100), IHC (1:500)
Goat anti-Rabbit IgG (H+L) 2nd Ab, HRP	Bio-Rad	1706515	WB (1:5000)
Goat anti-Mouse IgG (H+L)- 2nd Ab, HRP	Invitrogen	31430	WB (1:5000)
Goat anti-Rat IgG (H+L) 2nd Ab, Alexa Fluor Plus 488	Invitrogen	A11006	IF (1:100)
Donkey anti-Goat IgG (H+L) 2nd Ab, Alexa Fluor Plus 594	Invitrogen	A11058	IF (1:100)
Goat anti-Rabbit IgG (H+L) 2nd Ab, Alexa Fluor Plus 488	Invitrogen	A32731	IF (1:100)
Goat anti-Mouse IgG (H+L) 2nd Ab, Alexa Fluor Plus 488	Invitrogen	A11029	IF (1:100)

*CST, Cell Signaling Technology, USA.
